# Deciphering Short‐Range Order in 2D Transition Metal Dichalcogenides: From Origin to Multi‐Scale Property Modulation

**DOI:** 10.1002/advs.202524378

**Published:** 2026-04-17

**Authors:** Hanyu Liu, Linggang Zhu, Jian Zhou, Zhimei Sun

**Affiliations:** ^1^ School of Materials Science and Engineering Beihang University Beijing China

**Keywords:** chemical short‐range order, d‐band center, magnetic moment, mid‐gap state, transition‐metal dichalcogenide

## Abstract

Chemical short‐range order (SRO) is a critical structural characteristic in multi‐principal element materials, governing the global electronic properties including band gap. Yet its effect on site‐resolved properties, such as magnetic moments and d‐band centers, remains unclear. Here, using equiatomic 2D ternary transition metal dichalcogenides (TMDCs) exemplified by (V_0.5_Cr_0.5_)S_2_ and (Re_0.5_Ta_0.5_)S_2_, the origin and influence of SRO are unraveled via high‐throughput first‐principles calculations and machine learning. The results identify chemical affinity and atomic size difference as the dominant descriptors associated with SRO formation. Then, weak and strong SRO regimes are identified according to the energetic gain of SRO configurations relative to the quasi‐random states. Specifically, weak SRO in (V_0.5_Cr_0.5_)S_2_ has negligible influence on its half‐metallic character, but significantly modulates the site‐resolved properties including atomic magnetic moments and d‐band centers. Further, the mapping between the local atomic arrangements of (V_0.5_Cr_0.5_)S_2_ and the site‐resolved properties is accurately described by the many‐body descriptor MACE‐MP extracted from the universal interatomic potential. In contrast, strong SRO in (Re_0.5_Ta_0.5_)S_2_ suppresses the localized mid‐gap states originating from Ta_dz^2^ and Re_dz^2^/dx^2^‐y^2^ orbitals, leading to a semiconducting gap. These findings establish SRO as a fundamental degree of freedom for designing the multi‐scale functionalities of the materials.

## Graphical abstract

Short‐range order in 2D transition metal dichalcogenides is revealed as a new design paradigm. Driven by chemical affinity and atomic size, it governs properties across scales. Weak ordering tunes site‐resolved magnetism and d‐band centers, while strong ordering eliminates gap states to open band gaps. This work establishes short‐range order as a powerful strategy for functional materials design.

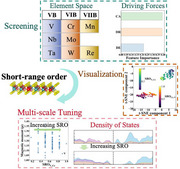

## 1 Introduction

2D transition metal dichalcogenides (TMDCs) have emerged as a prominent class of materials owing to their diverse crystal structures and tunable electronic, optical, and magnetic properties [1, 2, 3]. Their unique combination of semiconducting behavior [3], high carrier mobility [4], and strong spin‐orbit coupling [5, 6, 7] has enabled promising applications in nanoelectronics [8, 9], optoelectronics [10], catalysis [11, 12, 13, 14, 15], and various other domains. In particular, TMDC‐based catalysts have demonstrated significant potential for hydrogen evolution reactions [12] and CO_2_ reduction [11], highlighting their role in sustainable energy and environment application. Crucially, achieving precise control over their properties at the atomic scale is essential for optimizing performance in these sustainability‐related applications. In this regard, the physical and chemical properties of TMDCs are highly sensitive to subtle changes in the atomic‐scale chemical environment [16, 17], and thus element doping provides an effective means for tailoring the properties [18]. Conventional doping approaches typically introduce low‐concentration dopant, yet recent progress in multi‐principal element materials (MPEM), which incorporate high dopant concentrations, has revealed that compositional complexity can unlock novel physical properties unattainable in dilute doping regimes [19, 20].

For the atomic structure of MPEM, although random elemental distributions add a significant entropy contribution to the free energy at elevated temperatures [21, 22, 23], experimental and theoretical have revealed that local chemical fluctuations, chemical short‐range order (SRO) in particular, can persist at intermediate and low temperatures [24, 25, 26]. SRO has been widely observed across diverse MEPM from alloys [27, 28, 29] to electronic materials [30, 31], and represents an alternative degree of freedom for modulating mechanical and electronic properties. In the context of TMDCs, recent studies have unveiled the impact of SRO on their functional behavior. For example, in the monolayer (Re_0.5_Nb_0.5_)S_2_ alloy, SRO characterized by the preferentially neighboring of Re and Nb atoms against the random distribution, is observed, which substantially increases the bandgap compared to the case with random atomic distribution [32]. More importantly, this local ordering effect brings theoretical bandgap predictions into closer agreement with experimental measurements. Recently, the crucial role of SRO on the gap states engineering of quaternary Re_0.5_Nb_0.5_(S_0.5_X_0.5_)_2_ (X = Se, Te), is revealed, including the fine‐tuning of the gap states by the SRO in the non‐metal sublattice [33]. Beyond electronic structure, SRO‐induced variations in local chemical environments are also expected to play a critical role in catalytic performance. Recent study on multi‐component TMDCs has shown that site‐dependent hydrogen adsorption energies can vary substantially due to local ordering, and can be partially rationalized by descriptors such as the d‐band center, highlighting the potential of SRO as a handle for tuning catalytic activity [12].

However, pronounced SRO is not universal across alloyed TMDCs. For instance, in Mo‐W based alloys, SRO is weak and its influence on the properties is generally negligible [34]. Given the vast compositional space of alloyed TMDCs, identifying materials systems prone to forming SRO and elucidating the overall influence of SRO remain significant challenges. Moreover, current characterization of SRO primarily relies on pairwise structural descriptors, such as the Warren‐Cowley parameter, while descriptors that incorporate many‐body effects and directly link local structure to properties are highly desirable. Finally, exploring more potential influences of SRO on the materials properties beyond the band‐gap size is crucial for establishing SRO as an alternative degree of freedom in functional materials design. In this work, we aim at addressing these challenges regarding the SRO issues of TMDCs through a combination of first‐principles calculations and machine learning method.

## 2 Results and Discussion

### 2.1 High‐Throughput Investigation of SRO in Ternary TMDCs

**FIGURE 1:**
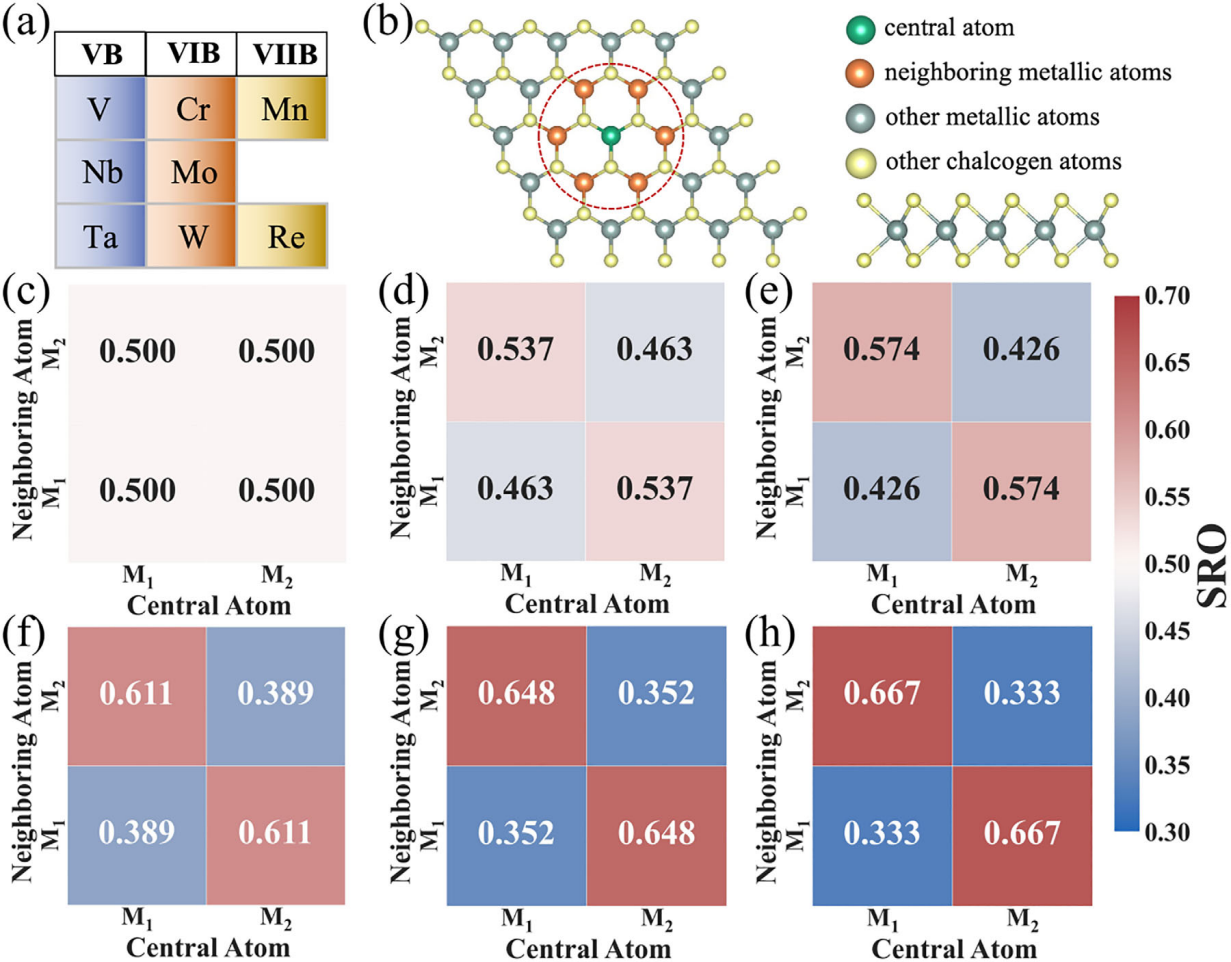
Alloying elements in high‐throughput screening, local atomic environments of TMDCs, and SRO parameters of six selected structures (SRO#1–SRO#6). (a) Selected elements for high‐throughput screening. (b) Top and side views of TMDC. The top view highlights the short‐range environment of a metallic atom. (c)–(h) SRO parameters for six representative (M_1_)_0.5_(M_2_)_0.5_S_2_ structures with varying degrees of SRO, labeled as SRO#1 to SRO#6, respectively.

In contrast to the negligible impact of SRO on the properties of (Mo_0.5_W_0.5_)S_2_ and (Mo_0.5_W_0.5_)Se_2_ reported by Yang et al. [34], Azizi et al. showed that SRO in monolayer H‐phase (Re_0.5_Nb_0.5_)S_2_ significantly affects its band gap [32]. This suggests that doping the transition metals from different groups (chemically dissimilar) is a promising strategy for identify TMDC systems in which SRO plays a critical role. To define the doping space, elements from group VB to VIIB of periodic table are selected, with technetium (Tc) excluded, as shown in Figure 1a. The atomic structure of H‐phase monolayer structure TMDC and local environment of the metallic atoms are illustrated in Figure 1b. In the present work, the analysis of SRO is restricted to the first nearest‐neighbor shell of the transition‐metal sublattice, where each metal atom is surrounded by six metal neighbors. Given the vast composition space of doped TMDCs, we restrict our study to the composition of (M_1_)_0.5_(M_2_)_0.5_S_2_ that exhibit a large configuration space, owing to equal concentration of transition metal M_1_ and M_2_. To characterize short‐range order across diverse configurations, a SRO parameter derived from Warren‐Cowley parameter is defined [35]:

(1) $$\mathrm{SR}O_{M_{1},M_{2}}=\frac{1}{K}\;\sum_{i\;=1}^{K}\frac{N_{M_{2}}}{N_{\mathrm{total}}}$$

where M_1_ and M_2_ denote the elements of the central and its neighboring element, respectively. K is the number of M_1_ atoms in the structure, $N_{M_{2}}$ is the number of neighboring M_2_ atom, N_total_ is the total number of the neighbor atoms surrounding the central atom M_1_. For the equiatomic (M_1_)_0.5_(M_2_)_0.5_S_2_ systems studied here, the $\mathrm{SR}O_{M_{1},M_{2}}$ value of less than 0.5 indicates that M_1_ and M_2_ avoid being neighbors with each other, whereas that of greater than 0.5 suggests a preference for M_1_‐M_2_ neighboring.

**FIGURE 2:**
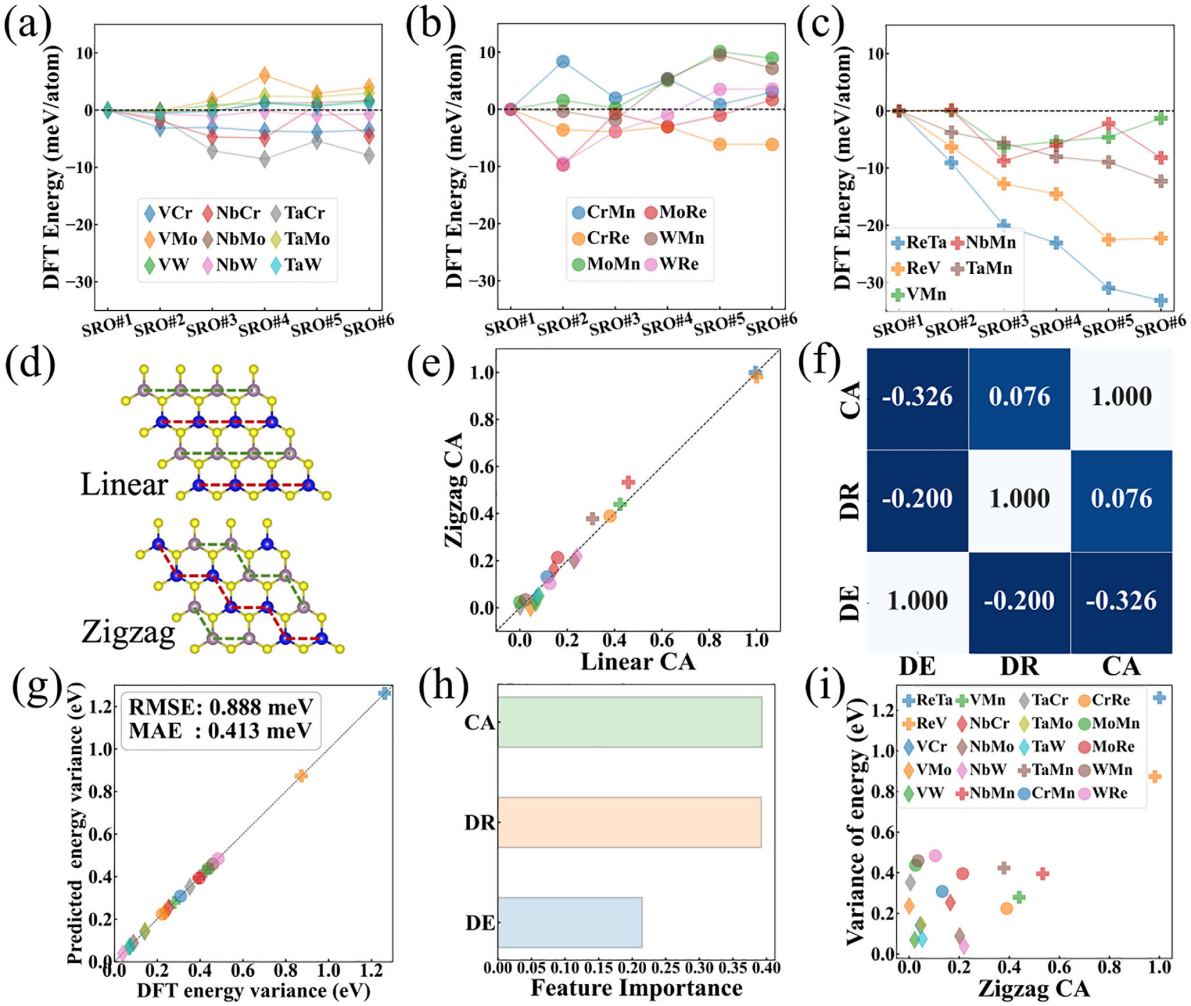
Relative energies of six sampled SRO configurations (SRO#1‐SRO#6) for different element combinations: (a) VB‐VIB, (b) VIB‐VIIB, (c) VB‐VIIB. (d) Two representative long‐range arrangements in H‐phase TMDCs: linear and zigzag patterns. (e) Comparison of linear chemical affinities and zigzag chemical affinities. Linear CA and Zigzag CA represent chemical affinity of linear and zigzag arrangements, respectively. (f) Pearson correlation coefficients among features. (g) DFT energy variance versus predicted energy variance. DE, DR and CA denote differences in electronegativity, atomic radius and chemical affinity of zigzag arrangement, respectively. (h) Feature importance scores from random forest model. (i) Correlation between zigzag chemical affinities and energy variance.

In our previous work [33], energy‐based Metropolis Monte Carlo (MC) simulations were firstly performed to sample the large configuration space induced by the different occupation sites of the metals in hexagonal (M_1_)_0.5_(M_2_)_0.5_S_2_ lattice. Six independent simulations were conducted at 923 K, each performing 1000 atomic swaps. Then here from the previous MC trajectories, six representative configurations with distinct and increasing SRO parameters from Figure 1c to h, labeled as SRO#1 to SRO#6, respectively, are chosen for further study. It can be seen that SRO#1 (Figure 1c) corresponds to the structure with the two metal elements distribute randomly in the metal sublattice of TMDC, while SRO#6 (Figure 1h) represents structures with rather ordered distribution of M_1_ and M_2_, which can be seen in Figure 2d. Structures with $\mathrm{SR}O_{M_{1},M_{1}}$ or $\mathrm{SR}O_{M_{2},M_{2}}$ larger than 0.5 (as shown in Figure S1), representing the clustering of the same type of elements, are not included in the present study. Although such configurations may be stabilized at elevated temperatures due to entropic effects, they tend toward phase separation at low temperatures [36]. It is noteworthy that for these six configurations picked from the extensive configuration space, their dimensionality‐reduced local atomic environment descriptors, detailed in Section 2.2, display a relatively continuous distribution in two‐dimension space, indicating that diverse local atomic environment have been effectively sampled. These six configurations will serve as the matrix for subsequent atom substitution.

Transition metal elements from different groups are combined (VB‐VIB, VIB‐VIIB, VB‐VIIB) and introduced to the six representative matrices (SRO#1 to SRO#6). Following structural relaxation, the relative energies of these systems with different SRO parameter are presented in Figure 2a–c, where the energy of each configuration is referenced to that of structure SRO#1 with a random arrangement of two metals. A negative energy value signifies that the system tends to exhibit short‐range order (SRO) with dissimilar metals preferentially forming nearest‐neighbor pairs. Conversely, a positive energy implies the system favors either a random atomic distribution or homoatomic clustering (phase separation), both of which are beyond the focus of the present work. Moreover, a monotonically energy decreasing trend from SRO#1 to SRO#6 with increasing SRO parameter represents a pronounced tendency for SRO in corresponding system. In VB‐VIB doped systems, the energy variation remains below 10 meV/atom. Notably, for systems including (V_0.5_Mo_0.5_)S_2_, (Nb_0.5_Mo_0.5_)S_2_, (Ta_0.5_Mo_0.5_)S_2_, (V_0.5_W_0.5_)S_2_, and (Ta_0.5_W_0.5_)S_2_, the corresponding energy is positive. Conversely (V_0.5_Cr_0.5_)S_2_, (Nb_0.5_Cr_0.5_)S_2_, (Ta_0.5_Cr_0.5_)S_2_ and (Nb_0.5_W_0.5_)S_2_ favor heteronuclear SRO. For VIB‐VIIB doped TMDCs, the corresponding energy curves basically oscillate around the reference line, indicating a lack of strong preference for SRO. Among the VB‐VIIB doped TMDCs, (Re_0.5_V_0.5_)S_2_, (Re_0.5_Ta_0.5_)S_2_ and (Ta_0.5_Mn_0.5_)S_2_, demonstrate a clear trend of decreasing relative energy with increasing $\mathrm{SR}O_{M_{1},M_{2}}$, suggesting that these systems energetically favor atomic arrangements where dissimilar elements are adjacent, implying strong heteroatomic ordering tendency. To quantitatively describe SRO in terms of energy, here we propose to use the energy variance across these six configurations (SRO#1‐SRO#6) as a proxy descriptor of the tendency to form SRO in the current systems, which is further used as the fitting target in the following machine learning study. A larger variance indicates that the system energy is more sensitive to variations in local atomic configurations, suggesting a stronger energetic preference for specific atomic arrangements.

In the study of SRO in high entropy materials, Chen et al. defined a so‐called chemical affinity (CA) [37] based on the cohesive energy, which is calculated as:

(2) $$\mathrm{CA}\left({\left(M_{1}\right)}_{0.5}{\left(M_{2}\right)}_{0.5}S_{2}\right)=\frac{E_{\max}\;-\;E_{c}\left({\left(M_{1}\right)}_{0.5}{\left(M_{2}\right)}_{0.5}S_{2}\right)}{E_{\max}\;-\;E_{\min}}$$

where E_c_((M_1_)_0.5_(M_2_)_0.5_S_2_) refers to the cohesive energy of a given (M_1_)_0.5_(M_2_)_0.5_S_2_ configuration with a specific atomic arrangement, while E_max_/E_min_ denotes the highest/lowest cohesive energy (E_c_) [37] across all considered elemental combinations. E_c_((M_1_)_0.5_(M_2_)_0.5_S_2_) for a given (M_1_)_0.5_(M_2_)_0.5_S_2_ configuration is calculated as:

(3) $$E_{c}\left({\left(M_{1}\right)}_{0.5}{\left(M_{2}\right)}_{0.5}S_{2}\right)=\frac{E_{\mathrm{tot}}\left({\left(M_{1}\right)}_{0.5}{\left(M_{2}\right)}_{0.5}S_{2}\right)-\sum_{M}n_{M}E_{M}^{\mathrm{atom}}}{N}\;$$

where E_tot_((M_1_)_0.5_(M_2_)_0.5_S_2_) is the total energy of the structure, $E_{M}^{\mathrm{atom}}$ is the energy of the isolated atom of element M, n_M_ is the number of atoms of type M, and N is the total number of atoms in the system.

In the practical computation of CA, for a specific composition, only configurations with predefined long‐range order is used to calculate the cohesive energy, i.e., the E_c_((M_1_)_0.5_(M_2_)_0.5_S_2_) term in Equation (2). Here, two distinct long‐range ordered structures, linear and zigzag arrangements (Figure 2d), are deliberately constructed as references for the monolayer H‐phase (M_1_)_0.5_(M_2_)_0.5_S_2_. The cohesive energies of these two long‐range ordered structures are listed in Table S1. It is noteworthy that the CA values obtained from the zigzag and linear configurations are nearly identical, as shown in Figure 2e. For simplicity, in the following discussion, CA values is those derived from the zigzag pattern. In addition to CA, differences of atomic radius (DR) and electronegativity (DE) can also contribute to the formation of SRO [38]. As shown in Figure 2f, the Pearson correlation coefficients among CA, DR and DE for the doped TMDCs are all less than 0.35, indicating weak correlations between these features that may contribute distinctly to the observed SRO tendencies. To further clarify how these features influence SRO, we construct a random forest regression model using CA, DR and DE to predict the energy variance among six representative SRO configurations (SRO#1∼SRO#6). A large energy variance implies that the energy of system changes significantly with varying SRO, and thus this energy variance can serve as an indicator of SRO tendencies. As shown in Figure 2g, the model achieves a RMSE of 0.888 meV/atom and MAE of 0.413 meV/atom, demonstrating that these features can effectively capture the energy fluctuations induced by SRO. Feature importance analysis in Figure 2h reveals that CA and DR contribute most strongly to the model, each with a feature importance of nearly 0.4. Meanwhile, DE also plays a meaningful role with an importance of about 0.2, suggesting that all these three descriptors are essential for capturing the energetic impact of SRO. Figure 2i reveals that the equiatomic VB‐VIIB TMDCs which exhibit strong SRO propensities, tend to be located in the upper‐right region relative to other systems, implying a positive correlation between CA and SRO. Previous theoretical investigations of Re‐Nb systems have demonstrated that SRO is strongly temperature‐dependent, as high‐temperature environments effectively disrupt SRO [33]. The experimental realization of SRO configurations is therefore intrinsically governed by thermal processing. As evidenced in medium‐entropy alloys, protocols such as controlled slow‐cooling or rapid quenching from annealing temperatures can kinetically freeze the system into specific energy‐minimized SRO regimes [39].

### 2.2 Weak SRO and Its Strong Effects on the Site‐resolved Properties of (V_0.5_Cr_0.5_)S_2_

**FIGURE 3:**
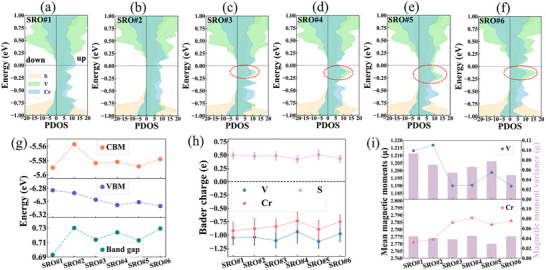
Electronic properties of SRO#1 to SRO#6 configurations in the (V_0.5_Cr_0.5_)S_2_ system. (a)‐(f) Projected density of states. (g) CBM, VBM and band gap for the spin‐down channel. (h) Error bar of charge transfer. (i) Mean and variance of atomic magnetic moments.

As shown in Figure 2a–c, alloyed TMDC systems may exhibit weak or strong SRO characterized by M_1_‐M_2_ pairing, which corresponds to a small or large energy decreasing with respect to the cases in which M_1_ and M_2_ are randomly distributed. In this section, (V_0.5_Cr_0.5_)S_2_ is selected as the representative system showing weak SRO, for further property study. Another reason for choosing (V_0.5_Cr_0.5_)S_2_ is its distinctive charge transfer behavior. As shown in Figure S2, the amount of charge transferred from V atoms in (V_0.5_Mo_0.5_)S_2_, (V_0.5_W_0.5_)S_2_, (V_0.5_Mn_0.5_)S_2_, and (V_0.5_Re_0.5_)S_2_ remains below 0.5 e, however, in (V_0.5_Cr_0.5_)S_2_, the charge transfer from V exceeds 0.9 e, indicating a substantially modified electronic environment. In addition, as shown in Figures S3–S5, the error bars of charge transfer for transition metals are generally small in most alloyed TMDC systems, indicating limited fluctuations among different atomic sites. In contrast, (V_0.5_Cr_0.5_)S_2_ exhibits a noticeably large error bar, suggesting significant site‐to‐site variation in charge transfer, rendering (V_0.5_Cr_0.5_)S_2_ an ideal system for probing the effects of SRO on atomic properties. The elastic modulus of six (V_0.5_Cr_0.5_)S_2_ configurations with different SRO are listed in Table S2, all of which satisfy the mechanical stability criterion (C11>0, C11*C22>C12*C12, det(Cij)>0) [40]. Among them, the lowest‐energy configuration ((V_0.5_Cr_0.5_)S_2_‐SRO#5) is further confirmed to be dynamically stable using AIMD simulation at 300K (Figure S6). The PDOS for the (V_0.5_Cr_0.5_)S_2_‐SRO#1 to (V_0.5_Cr_0.5_)S_2_‐SRO#6 are shown in Figure 3a–f, calculated by DFT+U method. All configurations exhibit a spin‐polarized half‐metallic character, with metallic behavior in the spin‐up channel evidenced by a finite density of states at Fermi level, and an insulating gap in the spin‐down channel, highlighting its potential for spintronic applications. As SRO_V,Cr_ increases, distinct peaks appears near the Fermi level in the spin‐up channel shown in Figure 3c–f, indicating a strengthened interaction between V and Cr atoms. Figure 3g presents the conduction band minimum (CBM), valence band maximum (VBM) and band gap values in the spin‐down channel for six configurations. As SRO increases, the CBM and band gap display similar variation trends, while the VBM shows a monotonic decrease. Notably, all these electronic parameters fluctuate within a narrow range (<0.05 eV), suggesting that these three fundamental electronic characteristics of (V_0.5_Cr_0.5_)S_2_ remain largely unaffected by changes in atomic ordering. In Figure 3h, the error bars representing bader charge transfer across (V_0.5_Cr_0.5_)S_2_‐SRO#1 to (V_0.5_Cr_0.5_)S_2_‐SRO#6 are illustrated. While the charge transfer associated with S atoms remains relatively invariant with varying SRO_V,Cr_, the average charge transfer for both V and Cr atoms exhibit considerable fluctuations. Moreover, significant charge‐transfer variance for the same type of element is observed within each configuration. Figure 3i shows the statistical analysis of atomic magnetic moments, including both the mean values and variances. Interestingly, V and Cr display opposite trends in their mean magnetic moments with varying SRO_V,Cr_, partially compensating each other and thereby lead to a constant total magnetic moment. Overall, the fluctuations in both charge transfer and magnetic moments reveal the critical role of SRO in modulating the site‐resolved properties of (V_0.5_Cr_0.5_)S_2_.

**FIGURE 4:**
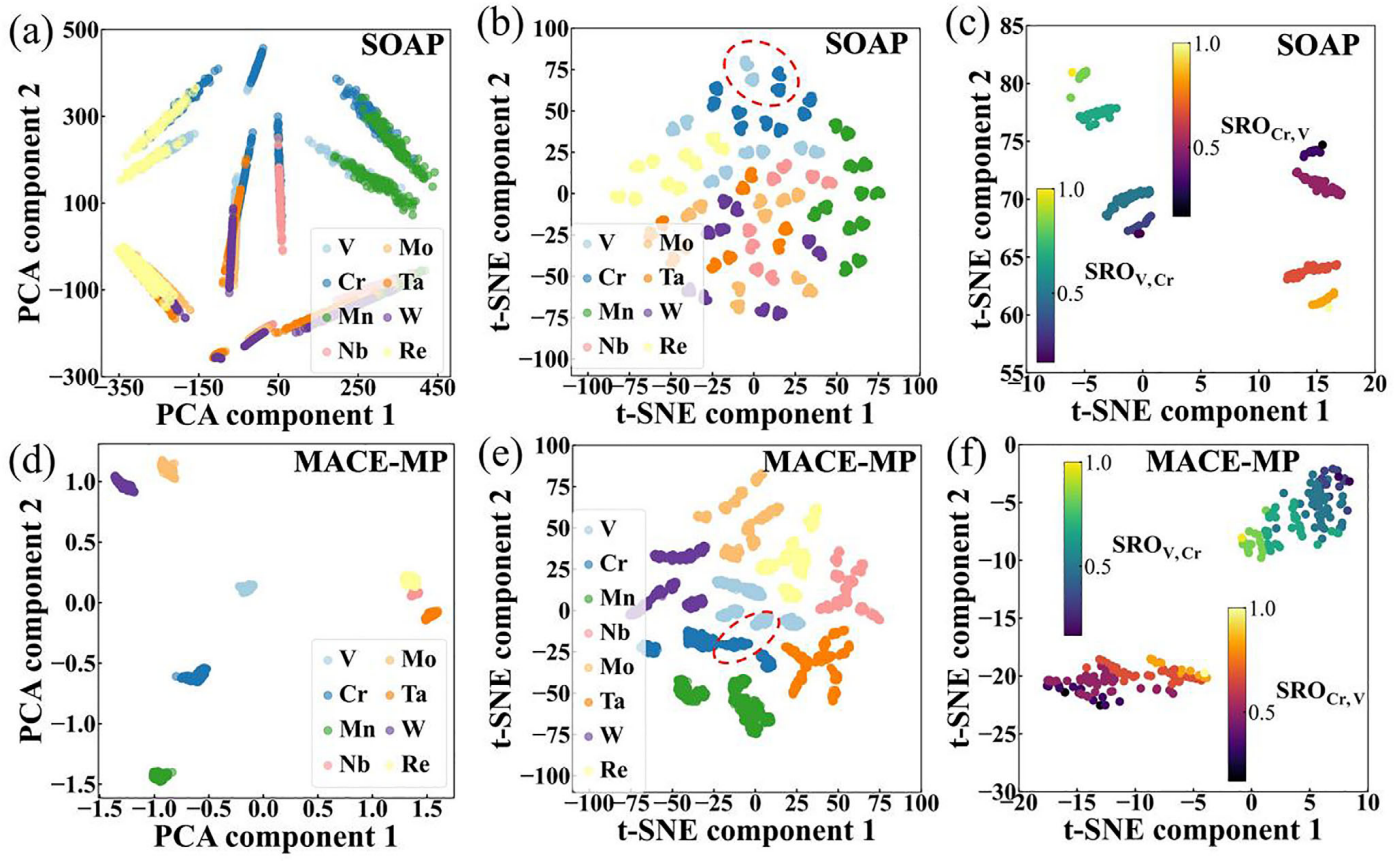
Dimensionality‐reduced representation of transition metal atom descriptors in all ternary TMDCs in the present work. (a)‐(b) SOAP descriptors reduced by PCA and t‐SNE. (c) t‐SNE projection of SOAP descriptors for V, Cr in (V_0.5_Cr_0.5_)S_2_, colored by SRO values. (d, e) MACE‐MP descriptors reduced by PCA and t‐SNE. (f) t‐SNE projection of MACE‐MP descriptors for V, Cr in (V_0.5_Cr_0.5_)S_2_, colored by SRO values.

Although the SRO parameter as calculated by Equation 1 explicitly quantifies the degree of SRO in the system, providing a clear measure of pairwise ordering, it is limited to element pairing preferences and serves only as a two‐body descriptor. To better characterize the local atomic ordering, especially to correlate the structural factor to site‐resolved properties, many‐body descriptors that go beyond two‐body correlations are necessary. Thereby, here the well‐known SOAP descriptor [41] is considered first, which captures both radial and angular distributions of neighboring atoms. The SOAP descriptors are generated using python package Dscribe [42], with cutoff radius set to 6.0 angstrom, and the number of radial basis functions to 8, the maximum degree of spherical harmonics to 6. Figure 4a and b display the dimensionality‐reduced SOAP descriptors using PCA and t‐SNE methods, respectively. As shown in Figure 4a, the PCA projection leads to significant overlap among data points corresponding to different elements, indicating limited capacity to distinguish atomic species. In contrast, the t‐SNE embedding in Figure 4b reveals well‐separated clusters, demonstrating superior resolution in capturing element‐specific features. Then the MACE‐MP [43] descriptor is further studied, which is derived from a pre‐trained MACE potential [43] based on MPtrj dataset [44]. Owing to its end‐to‐end architecture, the MACE‐MP descriptor provides a chemically informed and highly accurate representation of both elemental identity and local atomic environments. Figure 4d and f illustrate the dimensionality reduction of MACE‐MP descriptor via PCA and t‐SNE, respectively. Compared to SOAP (in Figure 4a and b), where data points corresponding to the same element may spread across different regions and points of different elements may overlap, MACE‐MP shows a clear clustering of points by element, with each element occupying a distinct region. This indicates that MACE‐MP provides better element discrimination than SOAP. As shown in Figure 4e, identical elements across various alloyed systems tend to cluster closely together, reflecting its robustness in capturing intrinsic chemical characteristics despite compositional variations. To further evaluate the capability of different descriptors in capturing SRO feature, the t‐SNE projections of SOAP and MACE‐MP descriptors are compared, using the property data of (V_0.5_Cr_0.5_)S_2_, as shown in Figure 4c and f. In both cases, atoms associated with distinct SRO are well‐separated, indicating that both descriptors are sensitive to SRO. However, SOAP‐based projection shown in Figure 4c yields a more discrete clustering of atoms with different SRO, whereas the MACE‐MP projection shown in Figure 4f exhibits smoother transitions between clusters. This difference likely stems from the nature of the descriptors: SOAP produces sparse representations akin to one‐hot encoding, where many components take zero values, while MACE‐MP leverages continuous type embedding, resulting in denser feature space, as illustrated by the descriptor visualization of (V_0.5_Cr_0.5_)S_2_‐SRO#1 in Figure S7.

**FIGURE 5:**
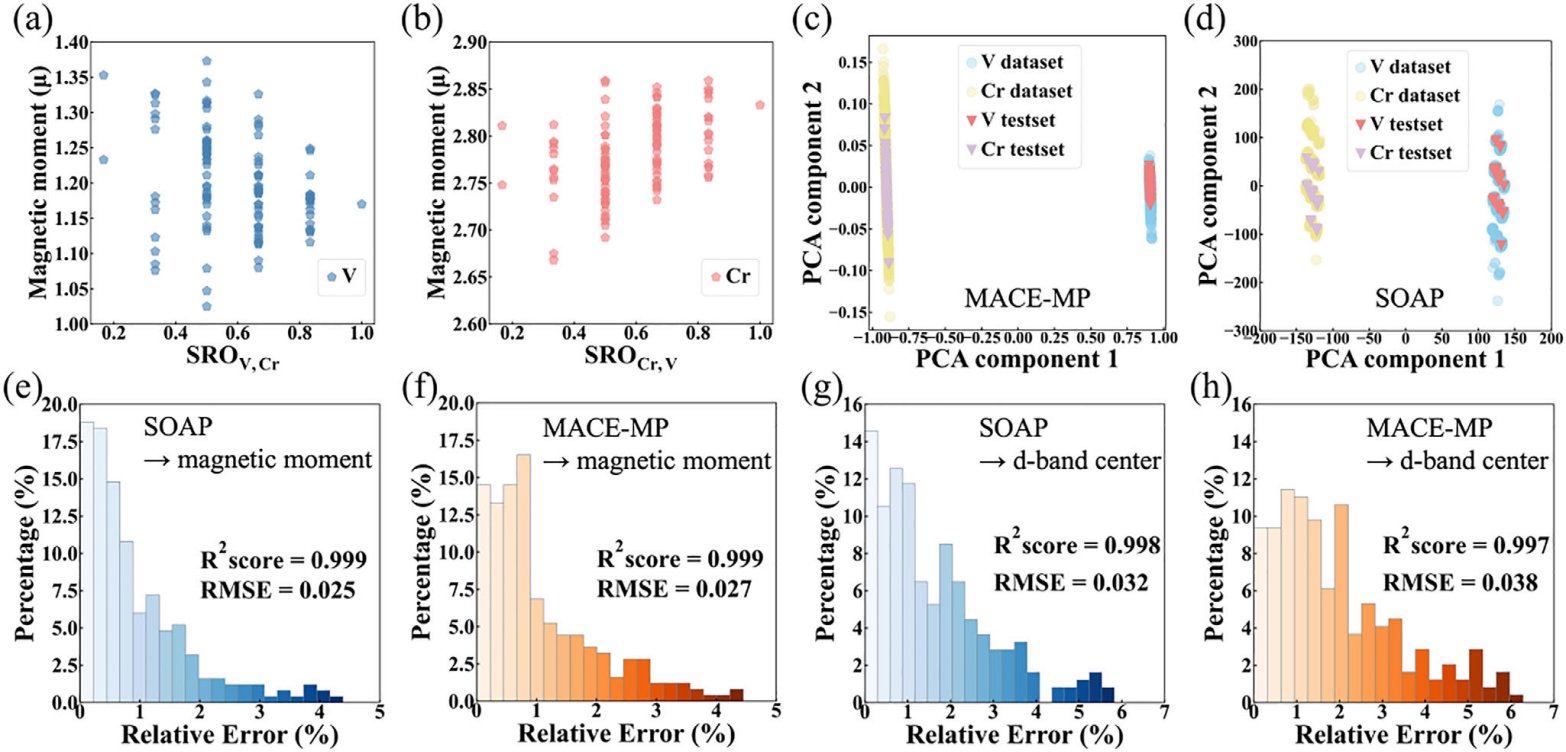
Relationships between atomic magnetic moments and short‐range order parameters, data distribution in descriptor space, and machine‐learning model performance for (V_0.5_Cr_0.5_)S_2_. (a) V atomic magnetic moments vs. SRO_V,Cr_. (b) Cr atomic magnetic moments vs. SRO_Cr,V_. (c) PCA projection of MACE‐MP descriptor for the full dataset and test set. (d) PCA projection of SOAP descriptor for the full dataset and test set. (e, f) Relative error distributions for SOAP‐ and MACE‐MP‐based model in predicting atomic magnetic moments. (g, h) Relative error distributions for SOAP‐ and MACE‐MP‐based model in predicting atomic d‐band center.

Previous analysis reveals that both the mean values and variances of V and Cr magnetic moment are sensitive to SRO parameters of (V_0.5_Cr_0.5_)S_2_ structures, and the following analysis is focused on establishing a mapping between the local atomic environment and site‐resolved properties of individual atoms including magnetic moment and d‐band center: magnetic moments are examined as indicators of magnetic response, while d‐band center is introduced as a widely recognized descriptor of catalytic activity [45], providing complementary insight beyond system‐averaged quantities. Figure 5a and b depict the distribution of atomic magnetic moments for all V and Cr atoms, respectively, as a function of SRO_V,Cr_ and SRO_Cr,V_ across the (V_0.5_Cr_0.5_)S_2_‐SRO#1 to (V_0.5_Cr_0.5_)S_2_‐SRO#6 configurations. In parallel, a corresponding analysis of d‐band centers distributions for the same sets of V and Cr atoms is provided in Figure S8. In Figure 5a, the magnetic moment of V atoms reaches a pronounced minimum of approximately 1 μ_*B*_ at SRO_V,Cr_ =  0.5, with a notable spread of values spanning nearly 0.4 μ_*B*_ at this point. Furthermore, when SRO_V,Cr_ deviates from 0.5 in either direction, the minimum V magnetic moment consistently exceeds 1.05 μ_*B*_. By contrast, the Cr atoms (Figure 5b) exhibit their smallest magnetic moment at SRO_V,Cr_ =  0.333. As shown in Figure 5a and b, a substantial scattering in the magnetic moments is observed even for atoms sharing identical SRO parameters values. Random forest models are constructed to map many‐body descriptors, SOAP and MACE‐MP, to site‐resolved properties. To capture finer details across diverse atomic environments, in addition to the 6 representative structures (SRO#1 to SRO#6), 64 additional structures are extracted at regular intervals from Monte Carlo trajectories reported in our previous work [33], followed by element substitution, resulting a total of 2520 atom‐property samples. The distribution of their SRO parameters is shown in Figure S9. The relative distribution of the (V_0.5_Cr_0.5_)S_2_‐SRO#1 to (V_0.5_Cr_0.5_)S_2_‐SRO#6 data points within the entire dataset is illustrate in Figure S10a and b. For model training, dimensionality reduction is first performed using PCA, followed by farthest point sampling (FPS) applied in the descriptor space of both MACE‐MP and SOAP to select diverse atomic environments. Such a strategy has been widely adopted in the development of interatomic potentials to ensure that the training set contains representative configurations [46]. The dataset is then split into training and test subsets at an 8:2 ratio. The distribution of the test set within the entire dataset is visualized in Figure 5c and d, while the corresponding training set distribution is presented in Figure S10c and d. The detailed hyperparameters for the random forest models are provided in Table S3. Figure 5e and f present the performance of random forest models in predicting atomic magnetic moments using SOAP and MACE‐MP descriptors, respectively, evaluated on the testset. Owing to the relatively narrow variation range of magnetic moments, both models achieve exceptionally high R^2^ score (0.999). Consequently, the relative error distribution provides a more discriminating view of model performance. The SOAP‐based model yields a higher proportion of low‐error predictions and a smaller RMSE (0.025), indicating superior predictive accuracy. Figure 5g and h illustrate the predictive performance for the atomic d‐band centers using SOAP and MACE‐MP, respectively. Similar to the magnetic moments results, the SOAP‐based model attains superior accuracy, achieving R^2^ score of 0.998 and RMSE of 0.032. Despite SOAP's superior prediction accuracy in the present study, it is important to note that MACE‐MP descriptor has its own merits: MACE‐MP is an end‐to‐end learned representation trained on MPtrj dataset, featuring a fixed dimensionality of 256 that remains constant regardless of the number of elemental species. In contrast, the handcrafted SOAP descriptor has higher dimensionality (2100 in this work) and its size scales rapidly with increasing elemental diversity, as shown in Figure S11. Therefore, in scenarios involving datasets with a larger variety of elements, models based on MACE‐MP are expected to demonstrate more pronounced performance benefits and scalability advantages over SOAP‐based approaches.

### 2.3 Strong SRO and the Gap States Tailoring in (Re_0.5_Ta_0.5_)S_2_

**FIGURE 6:**
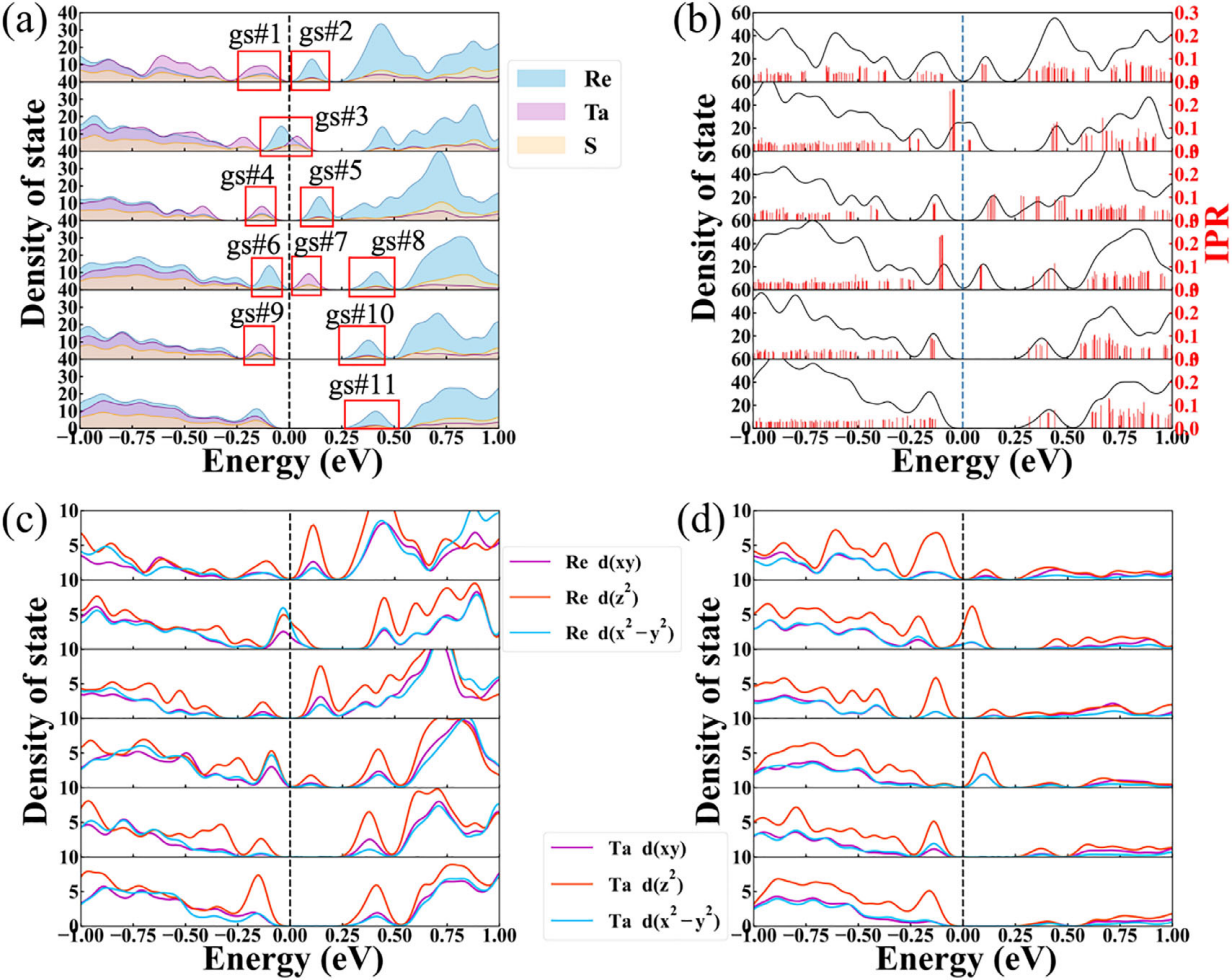
Electronic DOS for SRO#1‐SRO#6 configurations in the (Re_0.5_Ta_0.5_)S_2_ system. (a) Elemental PDOS. Gap states at various energy level are labeled as gs#1–gs#11. (b) Total DOS and IPR. (c) Orbital PDOS for Re. (d) Orbital PDOS for Ta.

In this section, (Re_0.5_Ta_0.5_)S_2_ that exhibits pronounced Re‐Ta SRO is investigated. Firstly, the mechanical stability of six (Re_0.5_Ta_0.5_)S_2_ configurations with varying SRO is confirmed by the elastic modulus in Table S4, while the dynamical stability of the lowest‐energy configuration (Re_0.5_Ta_0.5_)S_2_‐SRO#6) is further validated by AIMD simulation (Figure S12). As shown in Figure 6a, as SRO tendency increases from (Re_0.5_Ta_0.5_)S_2_‐SRO#1 to (Re_0.5_Ta_0.5_)S_2_‐SRO#6, the electronic structure varies from metal, semi‐metal to semiconductor, revealing a significant coupling between SRO and global electronic property. Moreover, at energies well below the Fermi level, the electronic states originate from both Re and Ta, representing a strong Re‐Ta interaction, while states well above the Fermi level are primarily dominated by Re. In particular, the electronic features in the vicinity of the Fermi level vary sensitively with SRO. For relatively disordered configurations, additional gap states appear within the band gap, mainly derived from Re and Ta orbitals. In more ordered configurations with larger SRO parameter, the gap states are suppressed, and only a Re‐derived contribution remains at the CBM, resulting in the opening of a distinct band gap. To quantify the degree of electron localization for the gap states, the inverse participation ratio (IPR) [47] is calculated as follows:

(4) $$\mathrm{IPR}\left(\psi_{n}\left({\vec{r}}_{i}\right)\right)=\frac{\sum_{i=1}^{N}{\left|\psi_{n}\left({\vec{r}}_{i}\right)\right|}^{4}}{{\left[\sum_{i=1}^{N}{\left|\psi_{n}\left({\vec{r}}_{i}\right)\right|}^{2}\right]}^{2}}\;$$

where N denotes the number of atoms in the configuration, and $\psi_{n}\left({\vec{r}}_{i}\right)$ represents the value of the nth orbital at the position of atom i. A higher IPR value indicates a more localized electronic state. In Figure 6b, generally, the Ta‐derived states exhibit a largely delocalized character, consistent across different configurations. By contrast, the Re‐contributed states show a stronger degree of localization, with particularly pronounced localization observed for gs#3 and gs#6. This different localization of Re and Ta states is consistent with the fact that d^5^ electron in Re is more localized than the d^3^ electron in Ta. The orbital PDOS of Re is shown in Figure 6c. Above the Fermi level, the dz^2^ orbital dominates the Re‐derived gap states, such as gs#2, gs#5, gs#8, gs#10, and gs#11. Below the Fermi level, the d(x^2^‐y^2^) orbital of Re contributes comparably to dz^2^, as observed in gs#3 and gs#6. Figure 6d presents the orbital PDOS of Ta. For all Ta‐derived gap states, the dz^2^ orbital exhibits a consistently dominant contribution compared to other orbitals. These DOS analysis highlight the crucial role of d orbital especially the dz^2^ in the chemical interactions in ternary TMDC.

**FIGURE 7:**
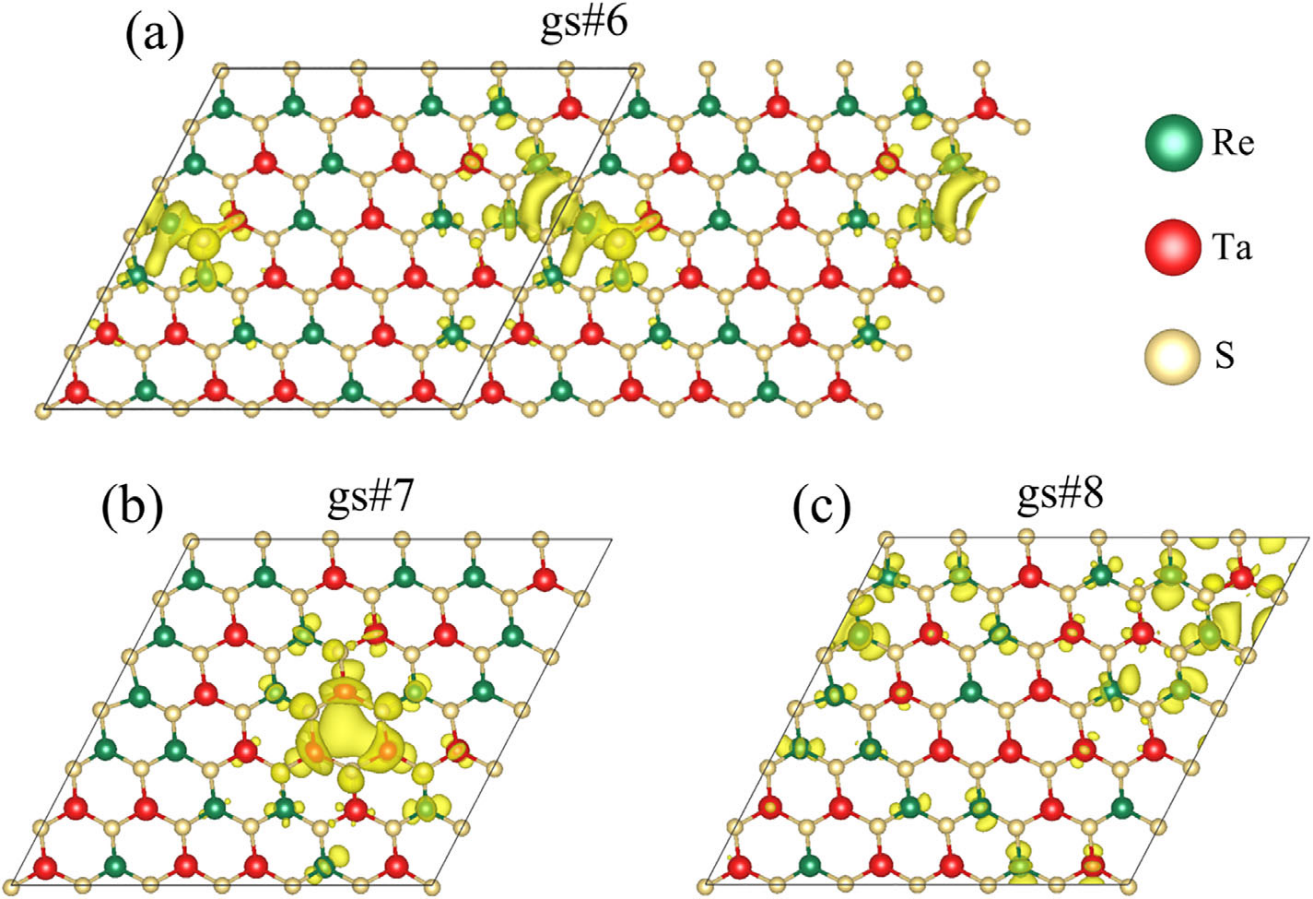
Partial charge density of gs#6, gs#7 and gs#8 gap states in (Re_0.5_Ta_0.5_)S_2_‐SRO#4 configuration, as labeled in Figure 6a. Isosurface values are set to be 0.001 e/Å^3^.

Building on the above analysis of the elemental and orbital contributions to the gap states, the origin of these gap states in terms of local atomic motifs are studied, aiming to establish a clear structure‐property mapping. Figure 7 shows the partial charge distribution (PCD) for various gap states, in configuration (Re_0.5_Ta_0.5_)S_2_‐SRO#4. The PCD for the remaining configurations are presented in Figure S13. The PCD corresponding to gap state gs#6 is shown in Figure 7a, revealing that gs#6 primarily originates from the Re cluster which comprises two planar triangular motifs. In Figure 7b, gs#7 is mainly associated with a Ta triangular cluster. Similar instances are observed in other configurations presented in Figure S13. gs#8 corresponds to the VBM of the (Re_0.5_Ta_0.5_)S_2_‐SRO#4 configuration and is primarily contributed by the dz^2^ orbital of Re. As shown in Figure 7c, gs#8 is associated with four‐ and five‐membered rings formed by Re atoms. Overall, the unpaired Ta and Re tend to generate the in‐gap states, and moreover, the gap states contributed by Re cluster normally located at a higher energy value than the states from Ta. When Ta‐Re pair forms, i.e, the SRO occurs, the Ta‐Re hybrid states will merge with the valence band and stay in lower energy level. Therefore, a strong coupling between local motif and the gap states can be observed in the current systems.

## 3 Conclusions

In this work, we conduct a high‐throughput screening of equiatomic alloyed H‐phase monolayer TMDCs incorporating elements from groups VB to VIIB, and demonstrate that their SRO tendencies are largely governed by chemical affinity and atomic size mismatch. Two representative materials systems, (V_0.5_Cr_0.5_)S_2_ and (Re_0.5_Ta_0.5_)S_2_, exhibiting weak and strong SRO, respectively, are selected for in‐depth analysis. In (V_0.5_Cr_0.5_)S_2_, after the formation of weak SRO, the spin‐polarized half‐metallic character of the system preserves, though stronger SRO enhances V‐Cr hybridization, producing a distinct spin‐up peak below the Fermi level. Although the global electronic structure of (V_0.5_Cr_0.5_)S_2_ is robust against local atomic arrangement, site‐resolved quantities such as atomic magnetic moments and d‐band centers vary substantially with local environments, which is accurately described using machine learning models with SOAP/MACE‐MP descriptors (with RMSEs of 0.025/0.032 μ_*B*_ and 0.027/0.038 eV for magnetic moments and d‐band centers, respectively). By contrast, (Re_0.5_Ta_0.5_)S_2_ exhibits strong SRO, where relatively random configurations lead to mid‐gap states originating from Ta_dz^2^ and Re_dz^2^/dx^2^‐y^2^ orbitals, while increased ordering suppresses these states and opens a clear bandgap. Overall, these findings not only clarify how SRO forms and governs the electronic properties of alloyed TMDCs, but also demonstrate that SRO provides a new degree of freedom for rational design of the multi‐scale functionalities of the materials.

## 4 Experimental Section

### 4.1 Ab Initio Calculations

First‐principles calculations were carried out within the framework of density functional theory (DFT) using the projector augmented wave (PAW) method as implemented in Vienna ab initio simulation package (VASP) [48, 49]. The exchange‐correlation interaction was treated using the generalized gradient approximation (GGA) parameterized by Perdew‐Burke‐Ernzerhof (PBE) [50]. A plane‐wave basis set with an energy cutoff of 500 eV was adopted to ensure the convergence of total energy. Structure relaxations were performed until the residual force on each atom was less than 0.01 $\mathrm{eV}\cdot {Å}^{-1}$ and the total energy variation was below 10^−6^ eV. The Brillouin zone was sampled using a k‐point mesh of 3 × 3 × 1 for geometry optimizations, and denser grids of 5 × 5 × 1 was employed for electronic property calculations. For monolayer H‐phase structures, all calculations were performed using a supercell containing 108 atoms. A vacuum layer larger than 20 Å was included along z‐axis to eliminate spurious interactions between periodic images. To better account for the localized nature of d electrons, the DFT+U [51] approach was employed for V and Cr atoms, with an effective Hubbard U parameter of 3 eV applied to both species [52, 53].

### 4.2 Machine Learning

To establish a quantitative mapping between local atomic environments and site‐specific properties, machine learning model was developed. The target quantities include the atomic magnetic moments and site‐resolved d‐band centers. Local structural environments were encoded using descriptors based on smooth overlap of atomic positions (SOAP) [41] and the MACE‐MP [43] from a pretrained model, both of which provides a high‐fidelity representation of short‐range order chemical and geometric correlations. A random forest regressor, as implemented in the scikit‐learn package [54], was employed to perform supervised learning.

## Conflicts of Interest

The authors declare no conflict of interest.

## Supporting information




**Supporting File**: advs75369‐sup‐0001‐SuppMat.docx.

## Data Availability

The data that support the findings of this study are available from the corresponding author upon reasonable request.
