# Supplementary material for: Deciphering Short‐Range Order in 2D Transition Metal Dichalcogenides: From Origin to Multi‐Scale Property Modulation
Source: Adv Sci (Weinh). 2026 Apr 17:e24378. Online ahead of print. doi: 10.1002/advs.202524378 (PMC13335446; doi:10.1002/advs.202524378)
Supplement: Supplementary file 1 — Supporting File: advs75369‐sup‐0001‐SuppMat.docx. [file ADVS-9999-e24378-s001.docx]

Supporting Information for

**Deciphering short-range order in two-dimensional transition metal dichalcogenides: From origin to multi-scale property modulation**

Hanyu Liu^1^, Linggang Zhu^1*^, Jian Zhou^1*^, Zhimei Sun^1*^

^1^School of Materials Science and Engineering, Beihang University, Beijing 100191, China.

*Corresponding authors:

lgzhu7@buaa.edu.cn, jzhou@buaa.edu.cn, zmsun@buaa.edu.cn


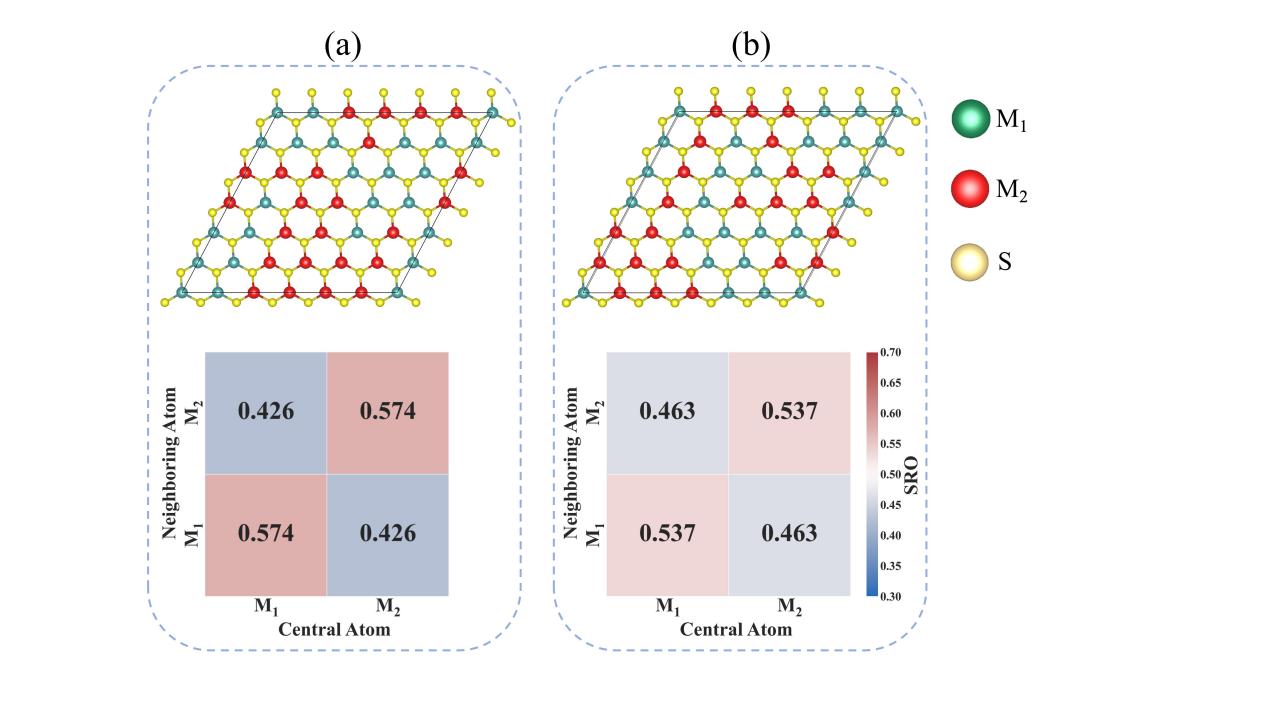


Figure S1. Two structures dominated by same-element clustering. (a) $\mathrm{SRO}_{M_{1},M_{2}}$=0.426. (b) $\mathrm{SRO}_{M_{1},M_{2}}$=0.463.

Table S1. The cohesive energy (unit: eV/atom) of ternary TMDCs. In these systems, the two metal atoms are arranged in an ordered pattern, including linear pattern and zigzag pattern, as displayed in Figure 2 (d).

| Composition | Linear pattern | Zigzag pattern |
| --- | --- | --- |
| (Re_0.5_Ta_0.5_)S_2_ | -0.700 | -0.726 |
| (Re_0.5_V_0.5_)S_2_ | -0.704 | -0.711 |
| (V_0.5_Cr_0.5_)S_2_ | 0.0478 | 0.047 |
| (V_0.5_Mo_0.5_)S_2_ | 0.0685 | 0.082 |
| (V_0.5_W_0.5_)S_2_ | 0.0512 | 0.065 |
| (V_0.5_Mn_0.5_)S_2_ | -0.238 | -0.273 |
| (Nb_0.5_Cr_0.5_)S_2_ | -0.011 | -0.050 |
| (Nb_0.5_Mo_0.5_)S_2_ | -0.080 | -0.081 |
| (Nb_0.5_W_0.5_)S_2_ | -0.090 | -0.095 |
| (Nb_0.5_Mn_0.5_)S_2_ | -0.265 | -0.349 |
| (Ta_0.5_Cr_0.5_)S_2_ | 0.105 | 0.078 |
| (Ta_0.5_Mo_0.5_)S_2_ | 0.039 | 0.043 |
| (Ta_0.5_W_0.5_)S_2_ | 0.042 | 0.040 |
| (Ta_0.5_Mn_0.5_)S_2_ | -0.143 | -0.223 |
| (Cr_0.5_Mn_0.5_)S_2_ | 0.012 | -0.024 |
| (Cr_0.5_Re_0.5_)S_2_ | -0.203 | -0.232 |
| (Mo_0.5_Mn_0.5_)S_2_ | 0.105 | 0.062 |
| (Mo_0.5_Re_0.5_)S_2_ | -0.024 | -0.090 |
| (W_0.5_Mn_0.5_)S_2_ | 0.087 | 0.054 |
| (W_0.5_Re_0.5_)S_2_ | 0.003 | -0.002 |


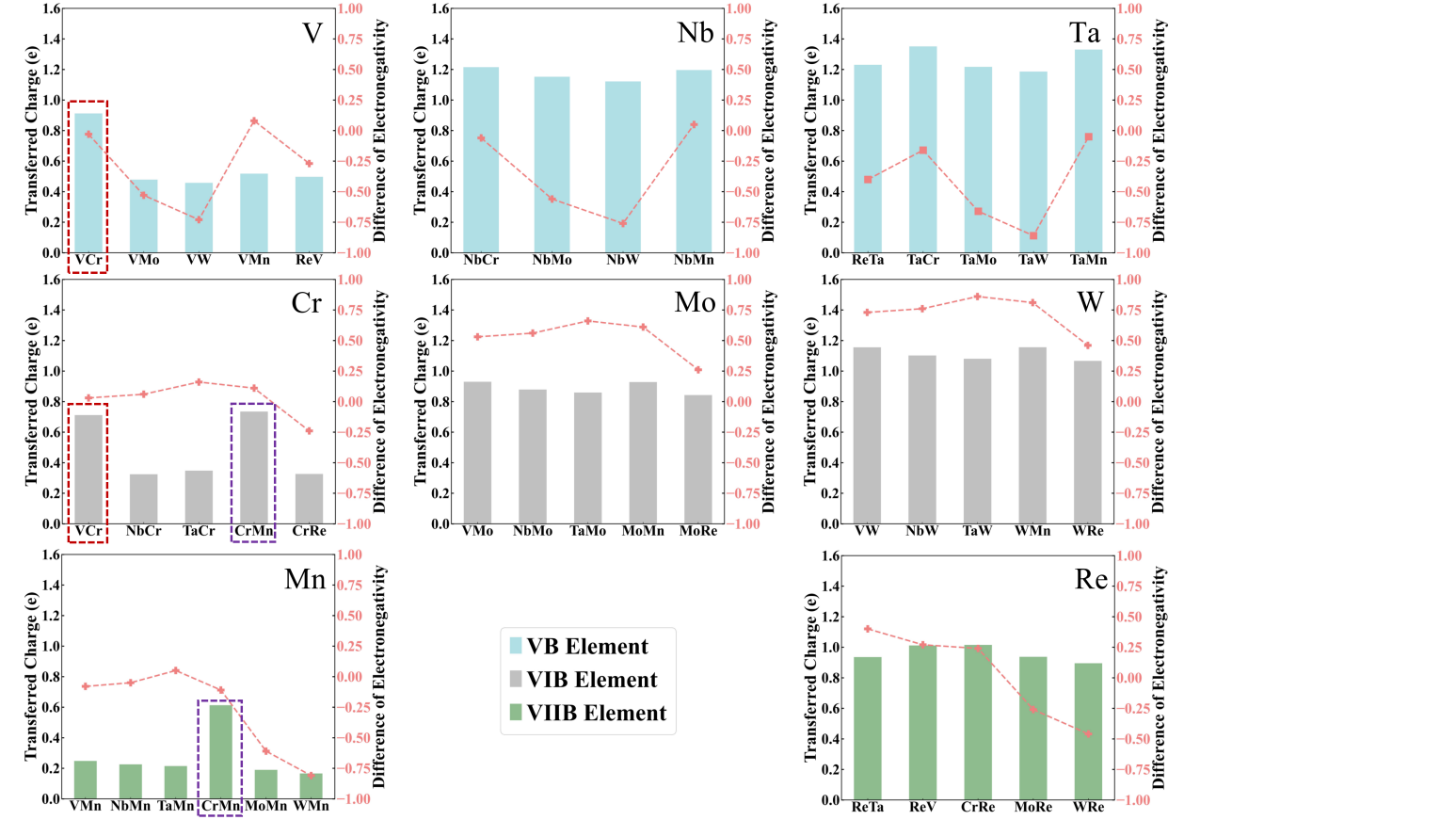


Figure S2. Average charge transfer and electronegativity difference of the metal atoms (V, Nb, Ta, Cr, Mo, W, Mn, Re) in (M_1_)_0.5_(M_2_)_0.5_S_2_ across six representative configurations (SRO#1 ~ SRO#6). Each subfigure corresponds to the data for one metal, for instance, the first subfigure shows the average charge transfer of V in the six configurations (SRO#1 ~ SRO#6) for each V_0.5_(M_2_)_0.5_S_2_ system, and the red line indicates the difference of electronegativity between V and the other metal M_2._


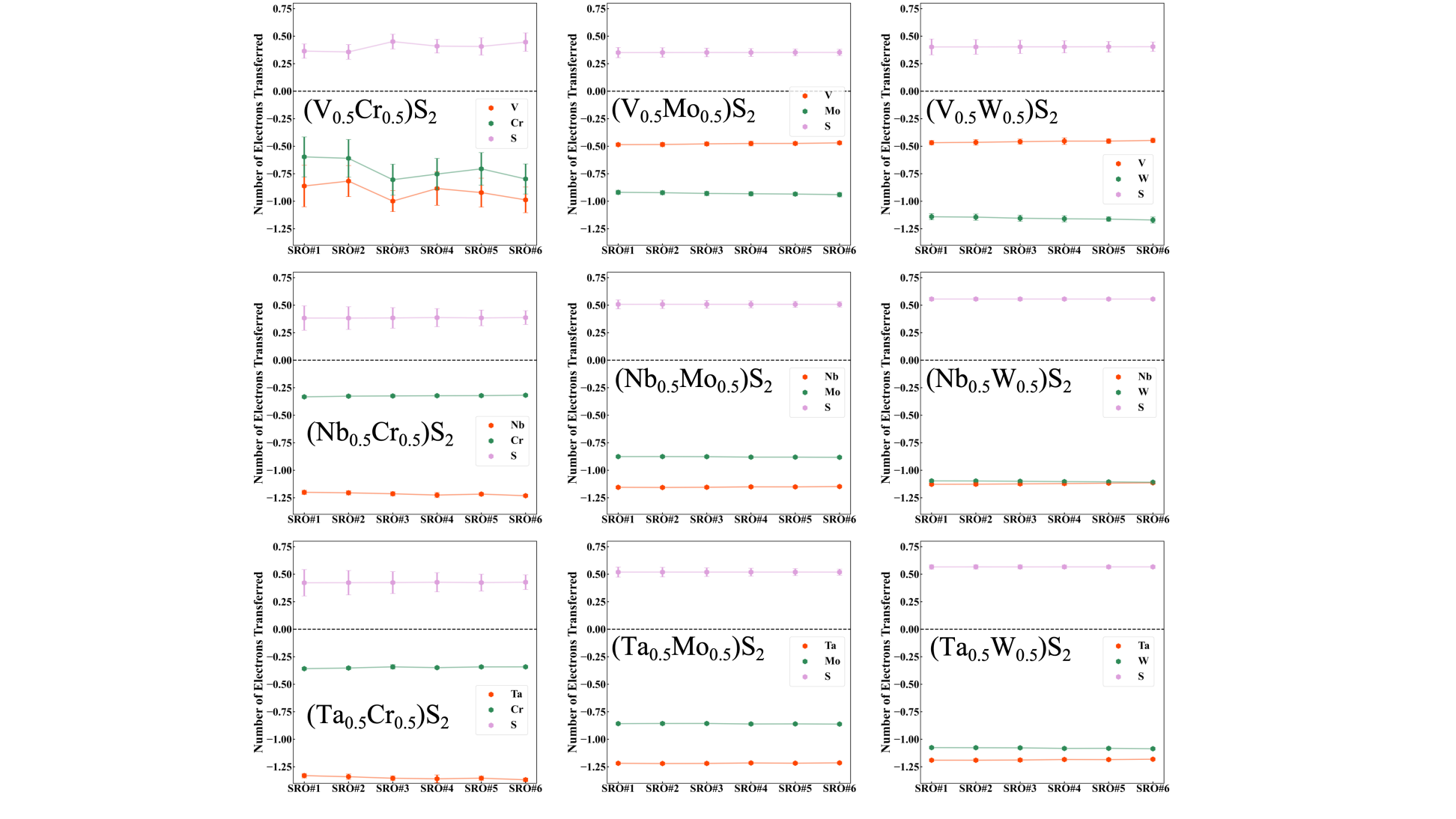


Figure S3. Plots of charge transfer for different elements in six representative SRO configurations (SRO#1~SRO#6) of (V_0.5_Cr_0.5_)S_2_, (V_0.5_Mo_0.5_)S_2_, (V_0.5_W_0.5_)S_2_, (Nb_0.5_Cr_0.5_)S_2_, (Nb_0.5_Mo_0.5_)S_2_, (Nb_0.5_W_0.5_)S_2_, (Ta_0.5_Cr_0.5_)S_2_, (Ta_0.5_Mo_0.5_)S_2_, and (Ta_0.5_W_0.5_)S_2_) .


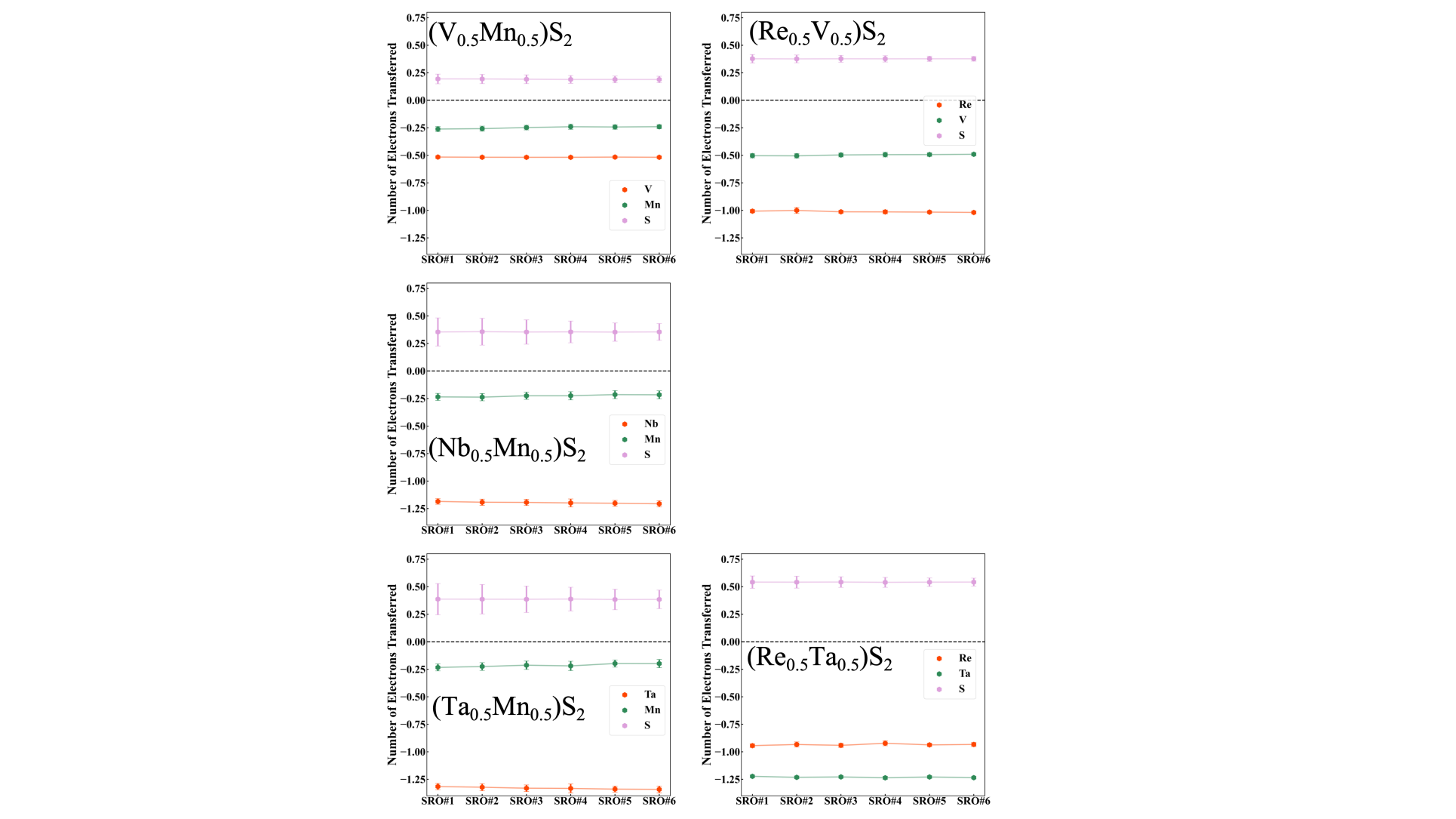


Figure S4. Plots of charge transfer for different elements in six representative SRO configurations (SRO#1~SRO#6) of (V_0.5_Mn_0.5_)S_2_, (Re_0.5_V_0.5_)S_2_, (Nb_0.5_Mn_0.5_)S_2_, (Ta_0.5_Mn_0.5_)S_2_, (Re_0.5_Ta_0.5_)S_2_.


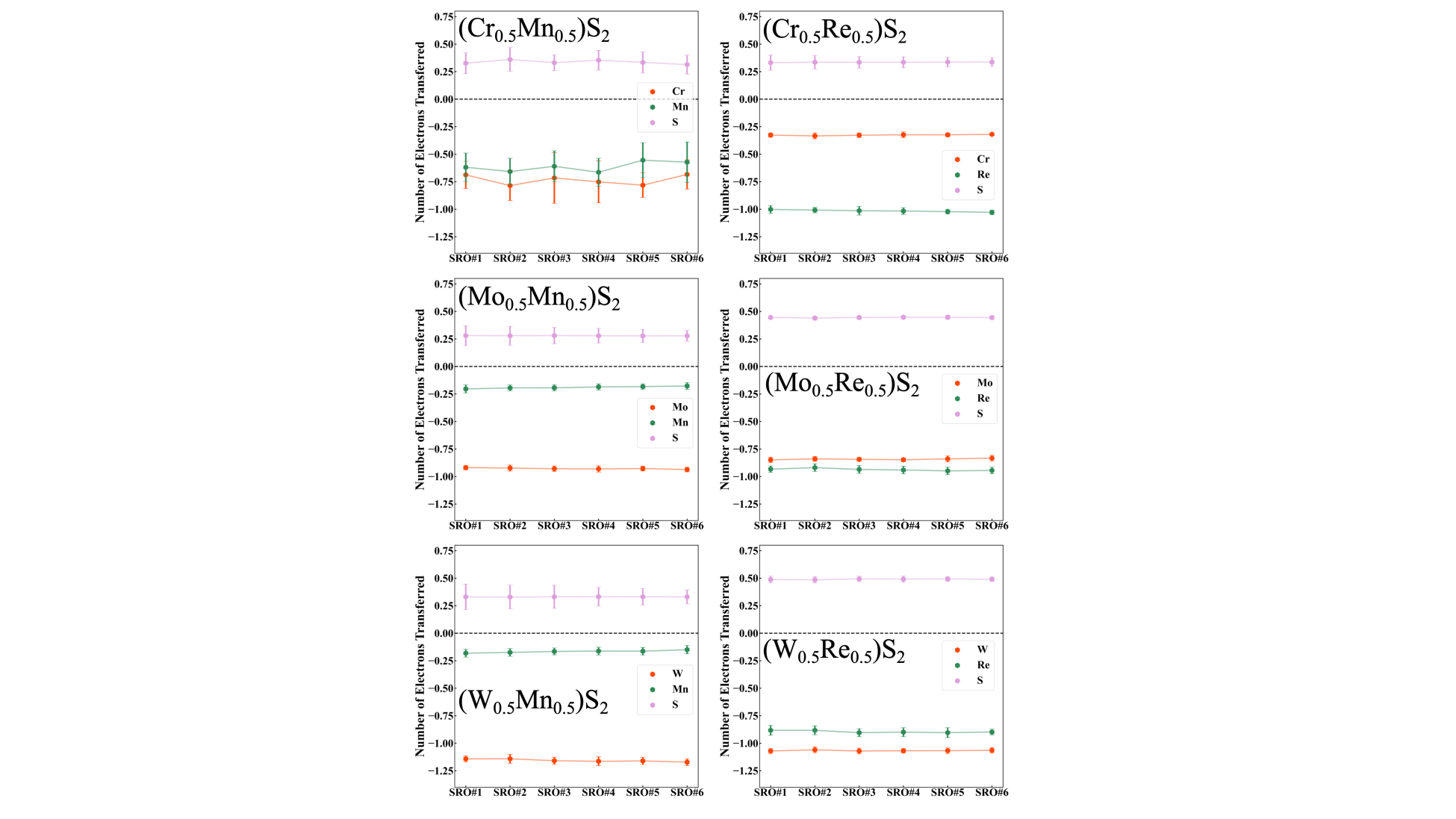


Figure S5. Plots of charge transfer for different elements in six representative SRO configurations (SRO#1~SRO#6) of (Cr_0.5_Mn_0.5_)S_2_, (Cr_0.5_Re_0.5_)S_2_, (Mo_0.5_Mn_0.5_)S_2_, (Mo_0.5_Re_0.5_)S_2_, (W_0.5_Mn_0.5_)S_2_, and (W_0.5_Re_0.5_)S_2._

Table S2. The elastic constants of (V_0.5_Cr_0.5_)S_2_-SRO#1 ~ (V_0.5_Cr_0.5_)S_2_-SRO#6 structures.

| Configuration | C11 | C12 | C16 | C22 | C26 | C66 |
| --- | --- | --- | --- | --- | --- | --- |
| (V_0.5_Cr_0.5_)S_2_-SRO#1 | 117.217 | 29.431 | 0.225 | 74.925 | 0.363 | 22.216 |
| (V_0.5_Cr_0.5_)S_2_-SRO#2 | 117.358 | 28.900 | -0.122 | 74.929 | 0.534 | 22.024 |
| (V_0.5_Cr_0.5_)S_2_-SRO#3 | 116.449 | 29.095 | -0.109 | 74.002 | 0.539 | 22.227 |
| (V_0.5_Cr_0.5_)S_2_-SRO#4 | 115.275 | 28.982 | -0.312 | 74.508 | 0.435 | 22.304 |
| (V_0.5_Cr_0.5_)S_2_-SRO#5 | 117.249 | 29.187 | 0.223 | 74.448 | 0.697 | 22.409 |
| (V_0.5_Cr_0.5_)S_2_-SRO#6 | 115.437 | 29.383 | 0.050 | 74.676 | 0.454 | 22.715 |


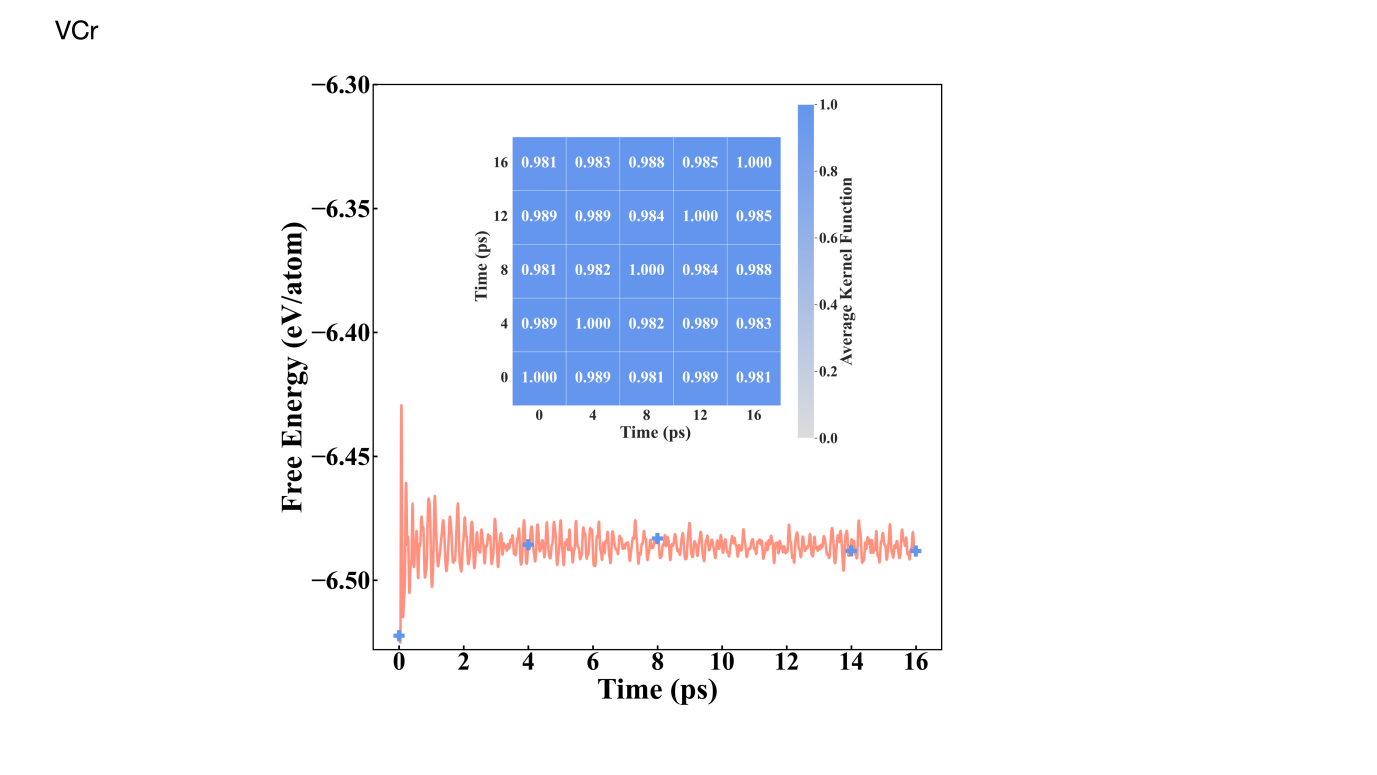


Figure S6. AIMD simulation at 300K for (V_0.5_Cr_0.5_)S_2_-SRO#5. The inset shows the similarity between structures obtained at 0ps, 4ps, 8ps, 12ps, and 16ps in AIMD simulation followed by a relaxation at 0K, which verifies the structure stability under annealing at 300K.


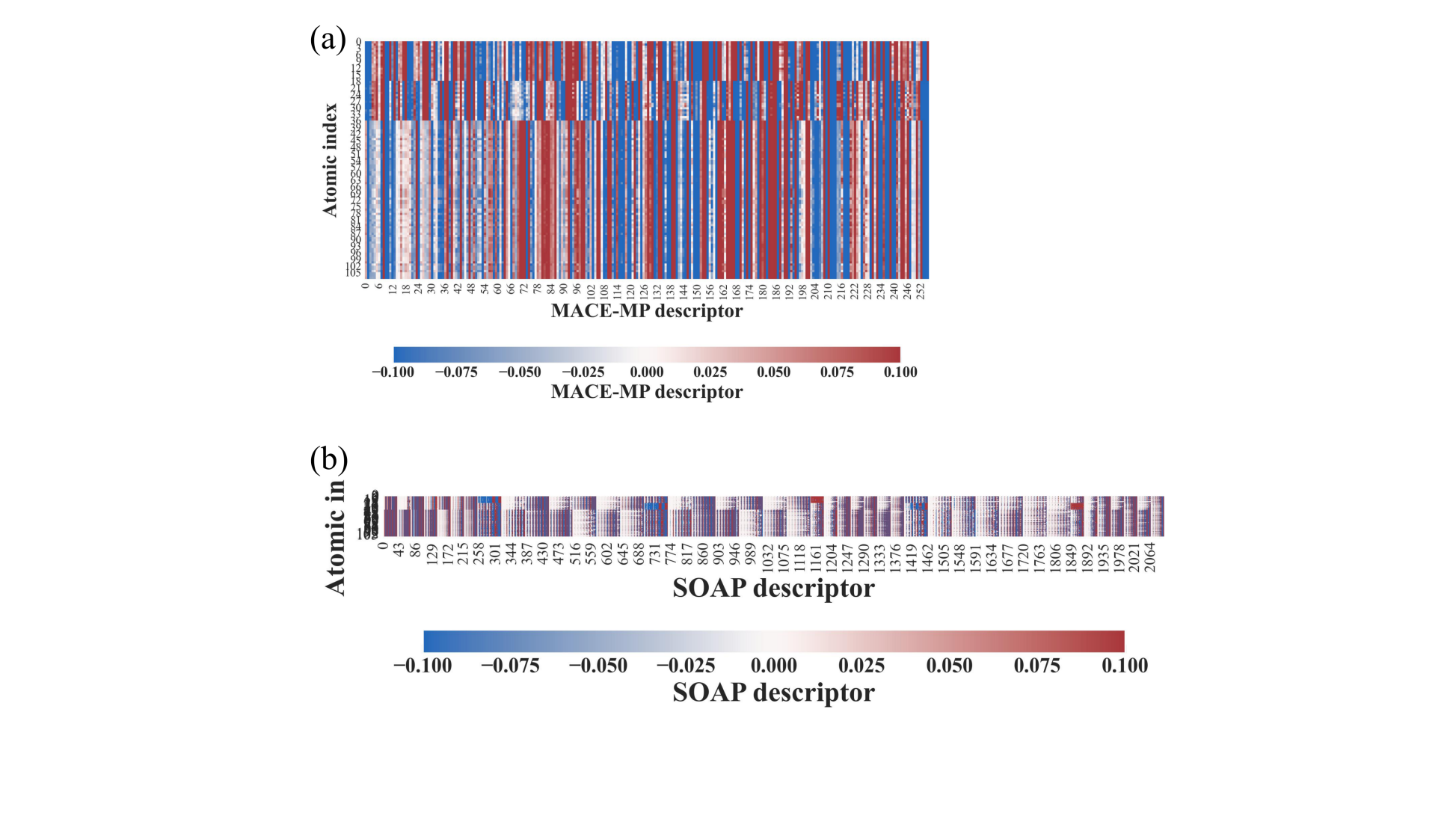


Figure S7. Descriptor visualization for configuration (V_0.5_Cr_0.5_)S_2_-SRO#1. (a) MACE-MP. (b) SOAP. A higher fraction of zero values indicates a sparser descriptor representation.


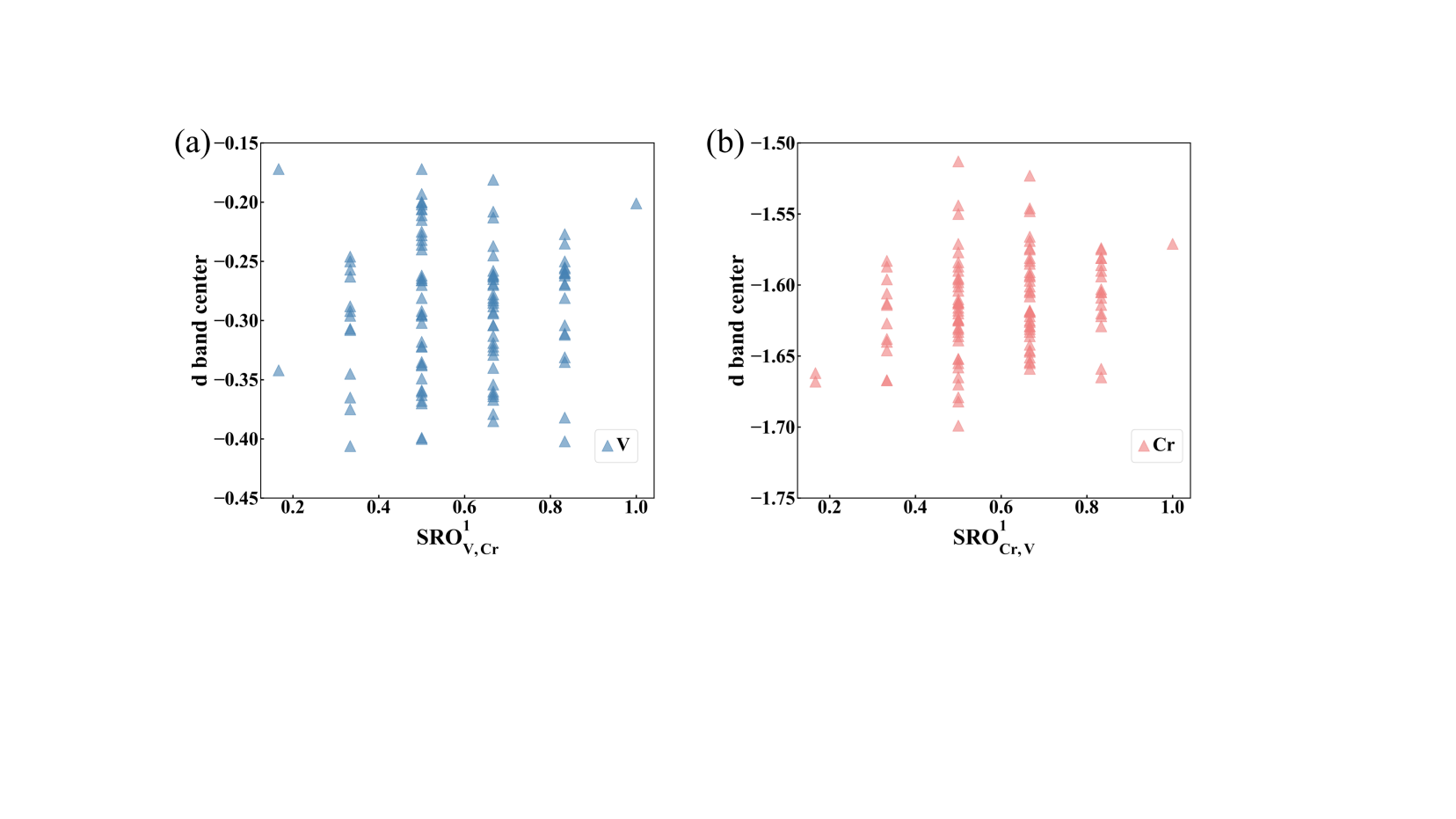


Figure S8. Relationship between atomic short-range order (SRO) parameters and d-band centers in (V_0.5_Cr_0.5_)S_2_, including the configurations SRO#1 to SRO#6. (a) V d-band center vs. $\mathrm{SRO}_{V,\mathrm{Cr}}$. (b) Cr d-band center vs. $\mathrm{SRO}_{\mathrm{Cr},V}$.


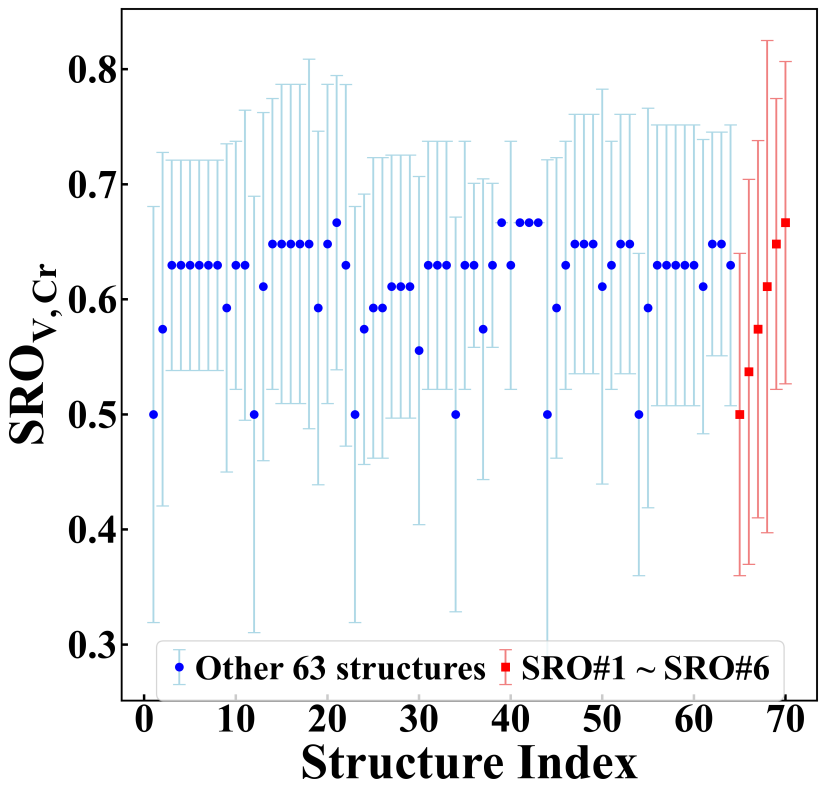


Figure S9. Mean and variance of $\mathrm{SRO}_{V,Cr}^{1}$ for all structures in the (V_0.5_Cr_0.5_)S_2_ machine learning dataset. The first 64 structures are sampled from six independent Monte Carlo trajectories, while the last six correspond to SRO#1–SRO#6 described in the main text. Some structures exhibit similar SRO parameters because the Monte Carlo simulations are nearly converged.


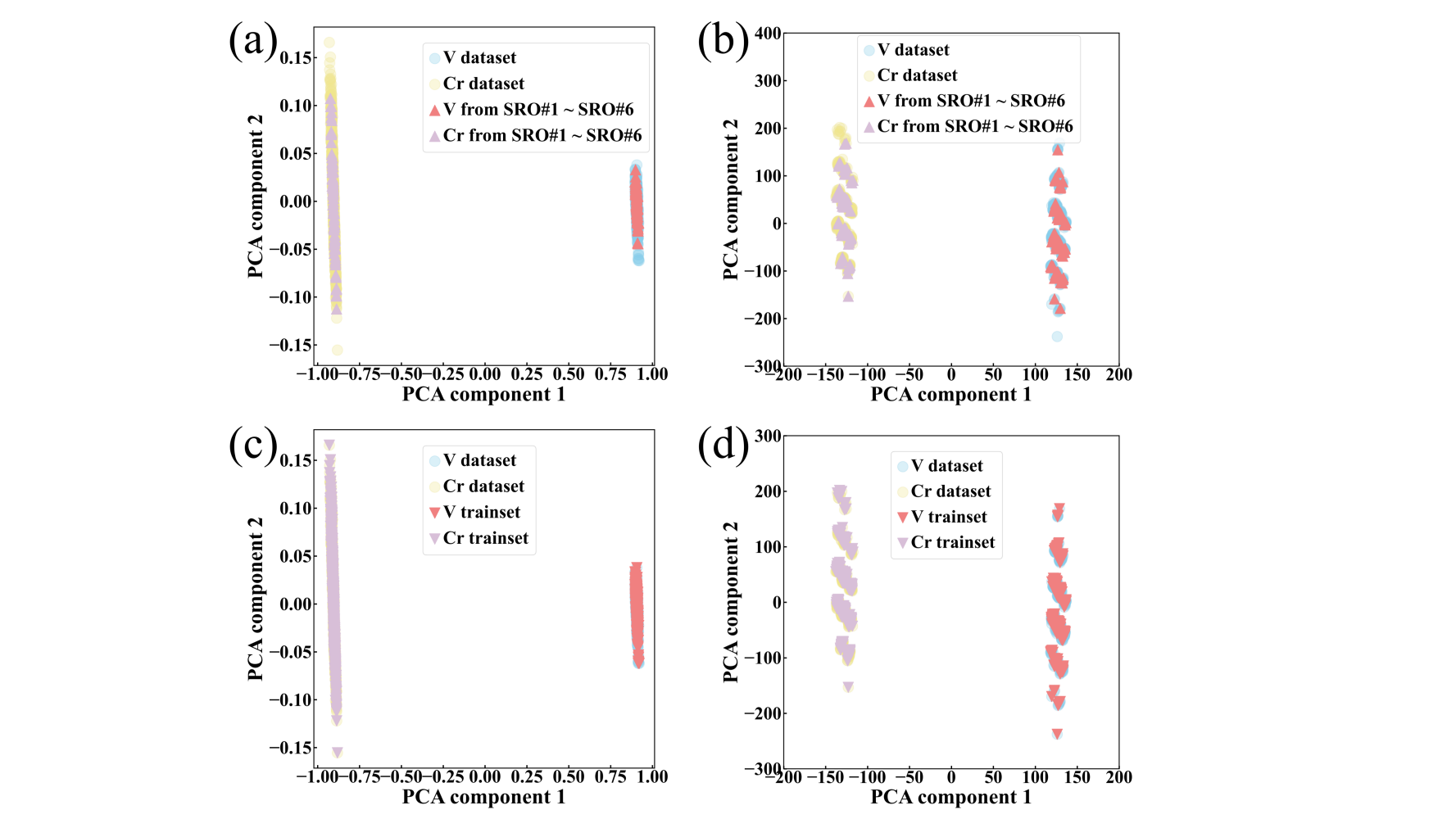


Figure S10. Visualization of data points distribution in dimension-reduced descriptor space. (a) PCA projection of MACE-MP descriptor for the full dataset and (V_0.5_Cr_0.5_)S_2_-SRO#1 to (V_0.5_Cr_0.5_)S_2_-SRO#6. (b) PCA projection of SOAP descriptor for the full dataset and (V_0.5_Cr_0.5_)S_2_-SRO#1 to (V_0.5_Cr_0.5_)S_2_-SRO#6. (c) PCA projection of MACE-MP descriptor for the full dataset and train set. (d) PCA projection of SOAP descriptor for the full dataset and train set.

Table S3. Hyperparameters of random forest model.

| max_depth | 11 |
| --- | --- |
| max_features | 0.55 |
| min_samples_split | 2 |
| min_samples_leaf | 1 |


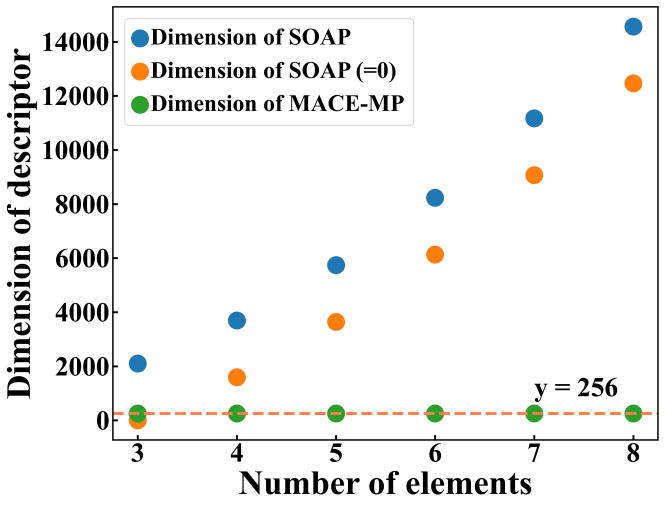


Figure S11. Dimension of descriptor vs. number of elements.

Table S4. The elastic constants of (Re_0.5_Ta_0.5_)S_2_-SRO#1 ~ (Re_0.5_Ta_0.5_)S_2_-SRO#6 structures.

| Configuration | C11 | C12 | C16 | C22 | C26 | C66 |
| --- | --- | --- | --- | --- | --- | --- |
| (Re_0.5_Ta_0.5_)S_2_-SRO#1 | 156.422 | 44.939 | 0.194 | 108.459 | -0.371 | 36.392 |
| (Re_0.5_Ta_0.5_)S_2_-SRO#2 | 159.484 | 44.112 | 0.438 | 113.575 | -0.279 | 37.631 |
| (Re_0.5_Ta_0.5_)S_2_-SRO#3 | 158.129 | 44.238 | -0.019 | 107.622 | 0.142 | 36.955 |
| (Re_0.5_Ta_0.5_)S_2_-SRO#4 | 160.487 | 44.267 | 0.031 | 113.663 | 0.176 | 37.980 |
| (Re_0.5_Ta_0.5_)S_2_-SRO#5 | 160.905 | 44.071 | -0.213 | 108.537 | 0.318 | 37.029 |
| (Re_0.5_Ta_0.5_)S_2_-SRO#6 | 160.119 | 43.929 | 0.116 | 109.258 | -0.104 | 37.228 |


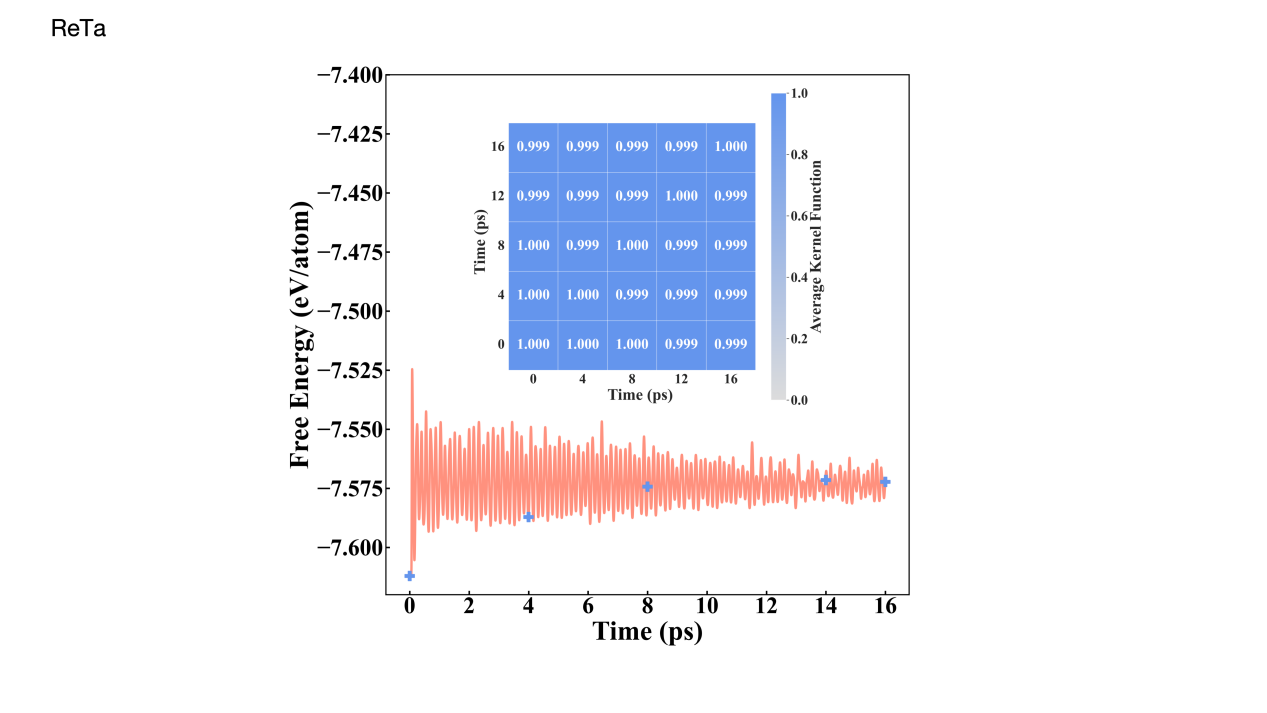


Figure S12. AIMD simulation at 300K for (Re_0.5_Ta_0.5_)S_2_-SRO#6. The inset shows the similarity between structures obtained at 0ps, 4ps, 8ps, 12ps, and 16ps in AIMD simulation followed by a relaxation at 0K, which verifies the structure stability under annealing at 300K.


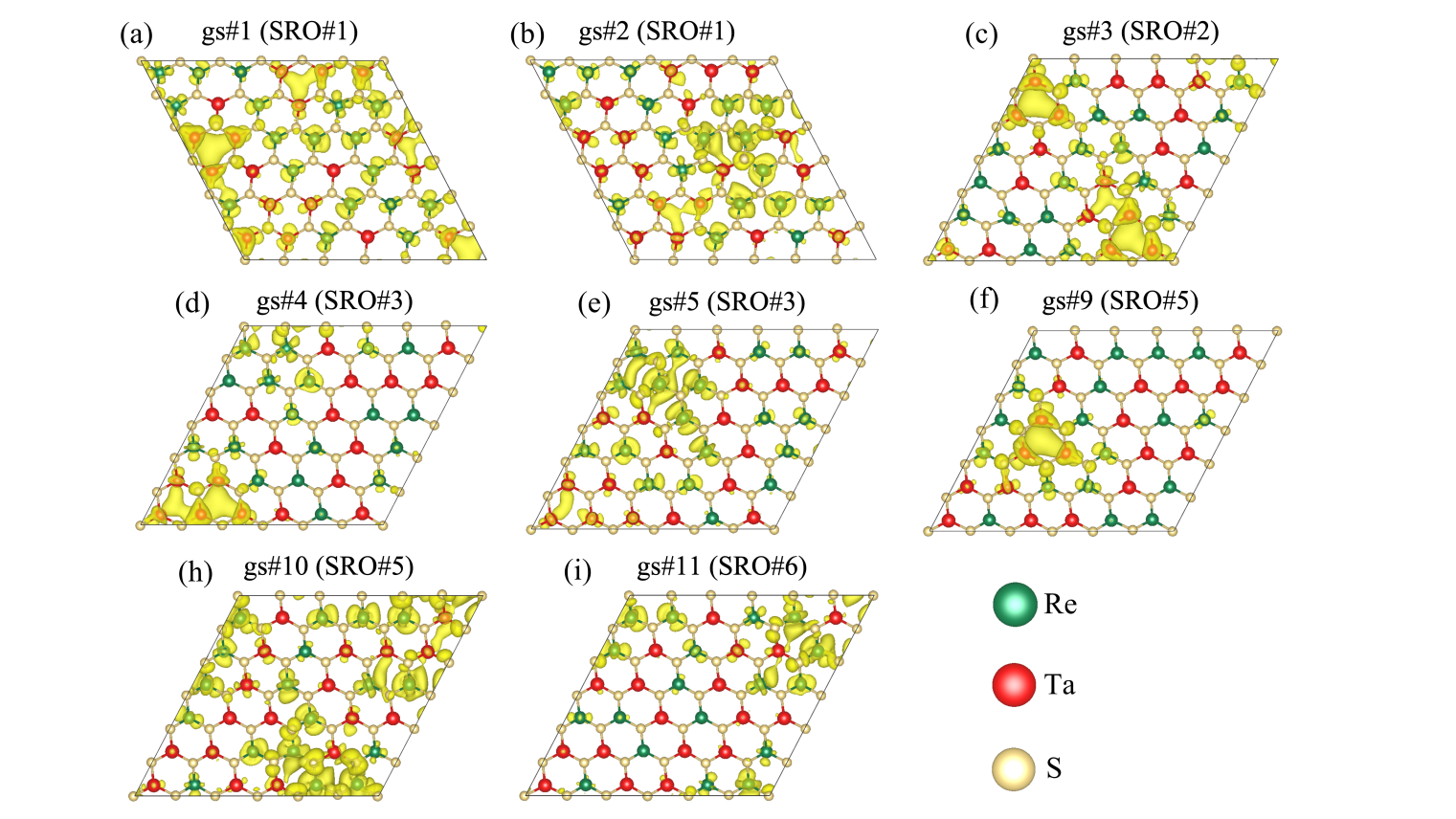


Figure S13. Partial charge density of gap states gs#1~gs#11 (except gs#6, gs#7, gs#8) as labeled in Figure 6 in the main text. Isosurface values are set to be 0.001 e/Å^3^.
